# Consequences of swamp forest fragmentation on assemblages of vascular epiphytes and climbing plants: Evaluation of the metacommunity structure

**DOI:** 10.1002/ece3.4635

**Published:** 2018-11-14

**Authors:** Jimmy Pincheira‐Ulbrich, Cristián E. Hernández, Alfredo Saldaña

**Affiliations:** ^1^ Laboratorio de Planificación Territorial Departamento de Ciencias Ambientales Facultad de Recursos Naturales Universidad Católica de Temuco Temuco Chile; ^2^ Programa de Doctorado en Sistemática y Biodiversidad Departamento de Zoología Facultad de Ciencias Naturales y Oceanográficas Universidad de Concepción Concepción Chile; ^3^ Laboratorio de Ecología Evolutiva y Filoinformática Departamento de Zoología Facultad de Ciencias Naturales y Oceanográficas Universidad de Concepción Concepción Chile; ^4^ Departamento de Botánica Universidad de Concepción Concepción Chile

**Keywords:** beta diversity, biodiversity, biogeography, ferns, landscape ecology, spatial autocorrelation

## Abstract

**Aim:**

Habitat reduction in fragmented landscapes provides an opportunity to study the biogeographic patterns that drive changes in diversity in poorly studied metacommunities. In this study, colonization–extinction dynamics were indirectly evaluated through the analysis of the species–area relationship and the nestedness of vascular epiphytes and climbing plants in 30 swamp forest fragments.

**Location:**

Coast of the Araucanía Region in Chile.

**Taxon:**

Vascular epiphytes (16 species, mainly Pteridophytes) and climbing plants (15 species).

**Methods:**

We used the database in Pincheira‐Ulbrich et al. (*New Zealand Journal of Botany, 54,* 2016, 458), where 904 trees were sampled and a total abundance of 41,097 fern fronds and 3,098 climbing stems were reported. For the species–area relationship, a simple linear regression model (SLR) and two models that consider the spatial autocorrelation of species richness among fragments, generalized least squares (GLS) and simultaneous autoregressive model (SAR), were compared. For the species nestedness, the nestedness measure based on overlap and decreasing fills (NODF) and weighted nestedness metric based on overlap and decreasing fill (WNODF) indexes were used on presence–absence and abundance matrices, respectively. These matrices were sorted by area size and distance from the largest fragment and then contrasted with the probability distribution of a randomized null model based on 10,000 simulations.

**Results:**

The results showed that the area size had a significantly positive effect on epiphyte species richness, while spatial autocorrelation played a fundamental role in explaining the richness of climbing plants. Both metacommunities had a general nestedness structure in terms of species incidence, which was determined first by area size and secondly by isolation.

**Main conclusions:**

Our results indicate that local colonization processes determined by species’ dispersal capacities could be the predominant mechanism for the spatial configuration of climbing plant species composition. On the other hand, selective extinction determined by patch size could characterize the spatial structure of epiphyte species’ composition.

## 1 INTRODUCTION

Vascular epiphytes and climbing plants are life forms that depend almost exclusively on other plants in order to survive (Benzing, 1990; Schnitzer, Bongers, Burham, & Putz, 2015). The problem with this close relationship is that changes in land use threaten the diversity of both plant groups in natural ecosystems, such as native forests. On a landscape scale, these land use changes lead to forest fragmentation, reduction and isolation, resulting in strong changes in diversity levels (e.g., Echeverría, Newton, Lara, Rey Benayas, & Coomes, 2007; Foley et al., 2005). These landscape‐scale processes can occur so fast that their consequences on the composition and structure of plant functional groups, such as climbing plants and vascular epiphytes, are not yet fully understood (Bartels & Chen, 2012; Campbell, Laurence, & Magrach, 2015). In addition, it is worrying that the current understanding of diversity changes in these plants is fundamentally based on the knowledge of descriptive‐observational patterns at the local level in most cases (e.g., Bartels & Chen, 2012; Campbell et al., 2015; Pincheira‐Ulbrich, 2011; Wagner, Mendieta‐Leiva, & Zotz, 2015). This limits our capacity to predict the effects of ecosystem reduction and fragmentation at landscape scale (see Gotelli & Colwell, 2001; Götzenberger et al., 2012; Ulrich & Gotelli, 2007).

In general, the reduction of habitat size has been identified as one of the major causes of species extinction because it is often directly related to a decrease in the population size of many species assemblages (Fahrig, 2003; Haddad et al., 2015; Tilman, May, Lehman, & Nowak, 1994). The fragmentation process reduces the core habitat while increasing the edge habitat; this determines new microclimatic conditions and new biological interactions that may change the structure of the original community (Gascon, Williamson, & Fonseca, 2000; López‐Barrera, Armesto, William‐Linera, Smith‐Ramírez, & Manson, 2007; Murcia, 1995). Forest loss and fragmentation may to affect epiphytes and climbing plants differently, since they constitute functional groups that are clearly distinguishable due to their morphology, physiology, life history (Bartels & Chen, 2012; Schnitzer & Bongers, 2002), and habitat specificity (Zotz, 2016). In temperate forests, for example, it has been demonstrated that climbing plants can explore habitat with different levels of canopy openness (Gianoli, Saldaña, Jiménez‐Castillo, & Valladares, 2010), while the moist conditions of the first few meters of the trunk provide a better microhabitat for epiphytic ferns (the more representative taxonomic group in temperate zones; Muñoz, Chacon, Perez, Barnert, & Armesto, 2003; Parra, Acuña, Corcuera, & Saldaña, 2009; San Martín et al., 2008; Woda, Huber, & Dohrenbusch, 2006), showing higher habitat specificity than climbing plants. However, in advanced stages of forest deterioration and with the loss of core areas, it is expected that many species disappear, depending on their ecological and physiological strategies in response to environmental filters (Campbell et al., 2015; Larrea & Werner, 2010; Schnitzer, 2005; Zotz & Bader, 2009). Although observations obtained at the local scale could contribute to landscape‐scale predictions, hypotheses that might explain their diversity response and current community structuring still need to be evaluated to generate specific predictions of the effects of human impact and the consequences of land use changes on richness and species assemblages (Bartels & Chen, 2012; Mohandass, Hughes, Campbell, & Davidar, 2014).

Although habitat loss in fragmented landscapes is a global problem, at the same time it offers an opportunity to test biogeographic hypotheses to explain diversity changes in poorly studied assemblages, such as occurs with assemblages of vascular epiphytes and climbing plants (e.g., Bartels & Chen, 2012; Campbell et al., 2015; Pincheira‐Ulbrich, 2011; Wagner et al., 2015). One classic hypothesis is the species–area relationship, where the number of species within a taxonomic group tends to increase within an increasing area (Connor & McCoy, 1979, 2001). Large areas would thus maintain species with more stable population sizes (lower probability of local extinction) and receive more immigration than small areas (higher probability of local extinction). This idea is not new, but did gain new importance within the framework of the theory of island biogeography (see Macarthur & Wilson, 2001). The emphasis in the species–area relationship is on predicting the species number and not on the taxonomical identity of those species (Macarthur & Wilson, 2001). Therefore, while fragment size may be a good predictor of richness, little can be inferred about the composition or structure of the assemblage in the metacommunity. It would therefore be of interest to evaluate the nestedness of the species composition within the same database (Ulrich, Almeida‐Neto, & Gotelli, 2009; Ulrich, Zalewski, & Uvarov, 2012; Almedida‐Neto & Ulrich, 2011).

The theory of island biogeography assumes that the balance between immigration and extinction rates should depend mainly on the size of the island and the distance from the mainland area—where habitat heterogeneity and functional traits are not considered important (Macarthur & Wilson, 2001). In a somewhat more complex approach, this balance could be represented by source–sink dynamics in a metacommunity where local communities in marginal areas (e.g., small fragments) can persist through immigration from nearby sources that are more productive or considered optimal habitats (e.g., large fragments; Leibold et al., 2004; Pulliam, 1988).

The source–sink metacommunity model assumes differentiated functional traits among species [e.g., tolerance to the matrix, ability to compete and sensitivity to disturbances (Ewers & Didham, 2005; Ulrich et al., 2009)], and habitat heterogeneity (e.g., density, taxonomic identity and different tree diameters), in such a way that richness and species composition within the landscape respond to this natural variation (Leibold et al., 2004). In this way, if area size produces nestedness and isolation does not, the system must be led by extinction because small patches have small population sizes, and colonization is therefore not sufficiently strong to generate nestedness. Under the opposite argument, that area size does not produce nestedness and isolation does, it is less clear whether it is selective immigration or extinction that determines the pattern (Bruun & Moen, 2003; Cutler, 1994; Lomolino, 1996; Ulrich, 2009; Ulrich & Gotelli, 2007), since local extinctions may actually be occurring but could be attenuated by a “rescue effect” from other fragments (Brown & Kodric‐Brown, 1977). In this way, the dispersal capacity of the species has an effect, accumulating species with high and low dispersal in nearby patches and only species with high dispersal capacity in distant patches (Dornier & Cheptou, 2012; Leibold et al., 2004).

In this context, the identification of idiosyncratic species (species that deviate from the general pattern of nestedness) may offer better possibilities for explaining biogeographic patterns (Ulrich et al., 2009). The particularity of these species is that they do not contribute to the general pattern of metacommunity nestedness, so they could be marking a differentiated response to the environmental gradient that is believed to drives this pattern. Consequently, the presence (or absence) of these species would not respond to the processes of colonization and extinction driven by the size and/or isolation of fragments. However, complete inventories are needed to identify idiosyncratic species and, in general, to properly infer the colonization and extinction dynamics at the landscape scale (Domínguez, Rebelo, & Bittman, 2012; Hortal, Lobo, & Jiménez‐Valverde, 2007; Rivera‐Huntiel, Bustamante, Marin, & Medel, 2012).

In nature, the species–area relationship and the nestedness of the species composition rarely fit perfectly to the expected model, which can be attributed mainly to both passive sampling and habitat heterogeneity (Connor & McCoy, 1979, 2001; Ulrich et al., 2009). In the passive sampling hypothesis, the more abundant species would more likely be found in large fragments purely by chance. This is due to the fact that metacommunities are typically characterized by species with highly unequal regional abundances that are distributed among patches, thereby larger areas are more likely to receive more propagules than small areas and these propagules are more likely to represent a wider array of species than the pool of species arriving to small areas (Connor & McCoy, 1979, 2001; Ulrich et al., 2009). The habitat heterogeneity hypothesis proposes that the greater the heterogeneity of the habitat (at local scale), the weaker the effect of any environmental gradient that may influence the structure of the metacommunity in the landscape (Ulrich et al., 2009).

Habitat heterogeneity is likely the most important component of the species–area relationship (Boecklen, 1986) and nestedness of the species composition (Mouquet, Millerm, Daufresne, & Kneitel, 2006). For example, the literature shows that the structure of the habitat alone is capable of explaining many of the population changes, without a strong correlation with the size of the fragments (Kalmar & Currie, 2006). Thus, structurally more complex and heterogeneous habitats could offer resources for the establishment of a greater number of species that could coexist at a local and regional scale (Jaña‐Prado, Celis‐Diez, Gutiérrez, Cornelius, & Armesto, 2006). This is why the study of biogeographic patterns tends to be ambiguous if environmental heterogeneity is not controlled. In nature, it is sometimes impossible to control certain structural variables within an area, so the selection of study sites could play a fundamental role in the interpretation of the assemblage type (Götzenberger et al., 2012).

One way to approach the problem of habitat heterogeneity is to focus on structurally more homogeneous forests. In this context, secondary forests offer the opportunity to test biogeographic hypotheses on a metacommunity scale, since the age and floristic structure of these forests tend to be less diverse than primary forests (Donoso, 1993). Swamp forests along the coast of the Araucanía Region of Chile, within the South American temperate ecosystem, represent remnants of secondary forests left by anthropogenic degradation, since they occupy soils with less value for agricultural and forestry activities (Möller & Muñoz‐Pedreros, 2014; Ramírez, Ferriere, & Figueroa, 1983; San Martín, Troncoso, & Ramírez, 1988). The peculiarity of these forests is that two tree species dominate the community [*Myrceugenia exsucca* O. Berg and *Blepharocalyx cruckshanskii* (Hook. & Arn.) (Ramírez et al., 1983)]. This forest is highly fragmented and grows exclusively on flat land within a biogeographic area limited by the Coastal Mountain Range (Peña‐Cortés et al., 2011). This “archipelago” of secondary forest patches can be assimilated to a source–sink model, since the fragmentation gradient and isolation tend to increase from large fragments of 936 ha (“mainland”) with a maximum separation distances among patches of 60 km (Peña‐Cortés et al., 2011; Pincheira‐Ulbrich, Hernández, Saldaña, Peña‐Cortés, and Aguilera‐Benavente, 2016).

In this study, we evaluated the species–area relationship and the nestedness of the species composition of vascular epiphytes and climbing plants in fragments of swamp forest in the Araucanía Region. Based on previous evidence from other fragmented habitats (e.g., Echeverría et al., 2007; Pincheira‐Ulbrich, Rau, & Peña‐Cortés, 2009; Pincheira‐Ulbrich, Rau, & Smith‐Ramírez, 2012; Pincheira‐Ulbrich et al., 2016), we hypothesized that (a) the fragment size would be positively related to the species richness; (b) both, the size and isolation of the fragments would explain the assemblage pattern of the metacommunity within the landscape (see Bartels & Chen, 2012; Schnitzer & Bongers, 2002); and (c) given that vascular epiphytes and climbing plants constitute clearly distinguishable functional groups (Bartels & Chen, 2012; Schnitzer & Bongers, 2002), both patterns (species–area relationship and nestedness) would be more marked in epiphytes than in climbers (see Mohandass et al., 2014; Pincheira‐Ulbrich et al., 2016; Taylor et al., 2016). One of the mechanisms that could explain this pattern is a metacommunity source–sink dynamic (Leibold et al., 2004), based on the fact that the smaller forest fragments are located in an agroforestry matrix, with a gradient (north‐southwest) from higher to lower anthropic use (see the maps in Peña‐Cortés et al., 2011). These habitats constitute marginal environments (sink habitats) that could be maintained by the arrival of propagules from larger, better‐conserved habitats (source of colonization). Thus, small fragments would sustain relatively few species given the higher extinction rates, while large fragments would maintain a greater species richness and lower local extinction rate (e.g., Leibold et al., 2004; Macarthur & Wilson, 2001).

## 2 MATERIAL AND METHODS

### 2.1 Study area

**Figure 1:**
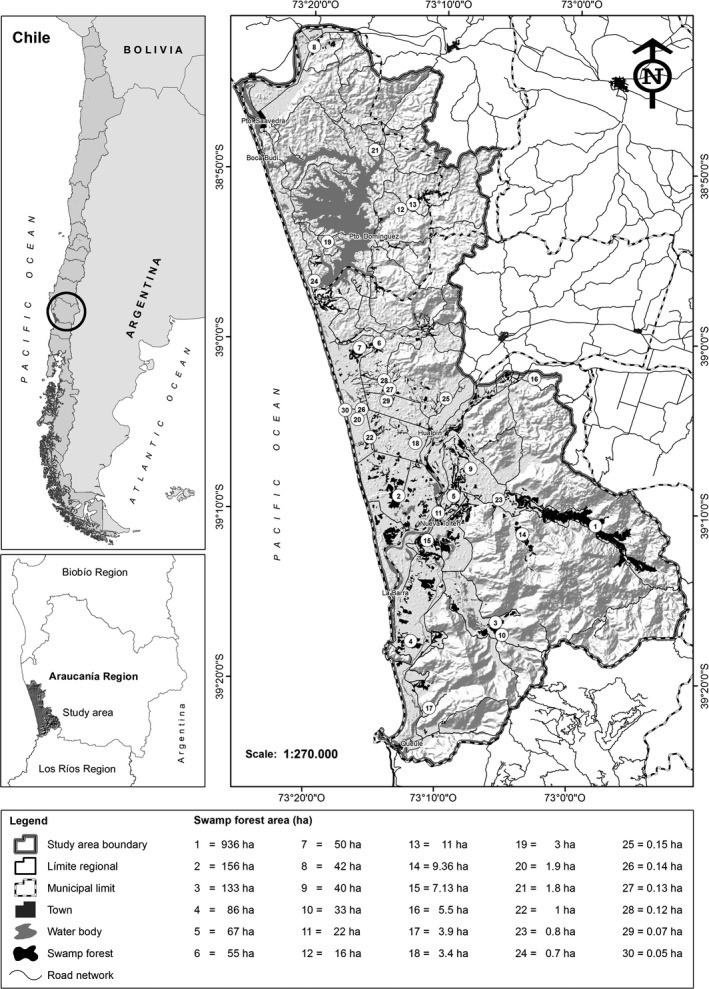
Map of the study area with the locations of the 30 swamp forest fragments along the coast of the Araucanía, Chile

The study area is part of the Araucanía Region of Chile's coastal forest remnants (Figure 1). It is located between 38°30′ and 39°30′S, and 72°45′ and 73°30′E, on the western side of the Coastal Mountain Range. The climate is oceanic with a Mediterranean influence (Luebert & Pliscoff, 2006) and average annual precipitations of 1,200–1,600 mm. The territory is characterized by an anthropic landscape extending from the mountain chain to platforms and terraces of marine abrasions and large fluviomarine plains where there are different types of wetlands (Peña‐Cortés et al., 2011, 2014).

Swamp forests are mainly located on temporarily or permanently waterlogged soils. This forest is principally composed of endemic species of the Myrtaceae family and is dominated by two species: *Myrceugenia exsucca* and *Blepharocalyx cruckshanskii* (Ramírez, San Martín, & San Martín, 1996; Ramírez et al., 1983). The swamp forest covers an area of 7,675 ha (4.6% of the territory), divided into 427 fragments of sizes varying between 923 m^2^ and 936 ha (Peña‐Cortés et al., 2011).

### 2.2 Database

The species richness and floristic composition of climbing plants and vascular epiphytes were obtained from Pincheira‐Ulbrich et al. (2016). In this research, 30 fragments of swamp forest were sampled (Figure 1) and the number of fronds and stems were, respectively, used as abundance measures. These surrogate measures are commonly used as criteria in clonal population studies (e.g., IUCN 2010; Mondragón, 2011; Wolf, Gradstein, & Nadkarni, 2009). In the case of the epiphyte *Fascicularia bicolor* (Ruiz & Pav.) Mez, the number of plants (rosettes) were recorded. The sampling design was nonrandom in order to include the greatest possible variety of microhabitats and rare species (Croft & Crow‐Fraser, 2009; Dieckman, Kühne, & Isermann, 2007). Sampling followed a ground‐based observation protocol (Flores‐Palacios & García‐Franco, 2001), using individual trees as the basic measure of each sampling effort. Trees are the quintessential substrate of these species, where plants are anchored and spend most of their life cycles. Trees were selected by transect sampling, oriented from the edge toward the center of each fragment (Brower, Zar, & Von Ende, 1990). In each transect, a circular quadrat of 3 m in diameter was established (7.06 m^2^), with a distance of at least 10 m between each quadrat. For logistical reasons (i.e., accessibility, cost and time), we sampled climbing plants and vascular epiphytes in 180 quadrats. The quadrats were established and georeferenced across the swamp forest with variable sampling intensities depending on the fragment size (site) and the accumulated species richness recorded in the field (Pincheira‐Ulbrich et al., 2016). In this way, a minimum of three quadrats were determined for small fragments (<1 ha) and a maximum of 18 quadrats for the largest fragment (936 ha). The sampling protocol resulted in 904 trees (min = 7, max = 87, $\overset{¯}{x}$ = 30 ± 18 trees in 30 sites) and reported a total richness of 16 epiphytes (mainly ferns with 41,097 fronds) and 15 native climbing plants (3,098 stems). The study of Pincheira‐Ulbrich et al. (2016) was essential for this research since it provided complete inventories at the metacommunity scale for both assemblages. This is strongly recommended to perform biogeographic studies of this type (Domínguez et al., 2012; Hortal et al., 2007; Rivera‐Huntiel et al., 2012).

### 2.3 Evaluation of the species–area relationship

The species–area relationship was evaluated by three regression models. These relate the area of the forest fragments (independent variable *X*) to the species richness of climbing plants and epiphytes (dependent variables *Y*), respectively (Dormann et al., 2007; Rangel, Diniz‐Filho, & Bini, 2006): (a) standard linear regression model (SLR), (b) generalized least squares model (GLS), and (c) spatial autoregressive model (SAR).

The GLS and SAR models explicitly use the UTM (universal transverse mercator) coordinates of the fragments. In practice, these models evaluate the effect of geographic space on species richness or, in other words, the effect that a set of fragments could have on one another: the neighborhood of fragments. This allowed us to assess the basic assumption that the residual errors in the regression model (*e*) are independent. Autocorrelation is expected to occur when nearby samples are more similar to one another than distant samples, as a consequence of a set of endogenous (e.g., dispersal) and exogenous (e.g., geomorphology) mechanisms that would explain the structure of the species distribution across the landscape (Kissling & Carl, 2008; Legendre & Fortin, 1989; Selmi, Boulinier, & Barbault, 2002).

In the standard linear regression model (in matrix notation: *Y *= *Xβ* + *e*), the slope vector (*β*) is calculated by the ordinary least squares: $\beta ={\left[X^{T}X\right]}^{-1}X^{T}Y$, where *X* ^T^ is the matrix transpose of *X* and superscript −1 is the inverse matrix (e.g., Quinn & Keough, 2002). Here, the residual error *e* was considered independent among observations (errors are homoscedastic), so that space would have no effect on species richness. On the other hand, in the GLS and SAR models, the residuals were not considered independent (errors are heteroscedastic), so the spatial autocorrelation was taken into account in two ways, respectively. In the case of the GLS model, the estimator of the slope vector ($\beta ={\left[X^{T}C^{-1}X\right]}^{-1}X^{T}C^{-1}Y$) incorporated the spatial structure into the model's residual directly into the variance–covariance matrix (C). To do this, Matrix C was modeled using a semivariogram, fitted by the spherical model (see Legendre & Legendre, 1998) based on visual inspection of the behavior of *Y* among all pairs of fragments (*y*_*j*_−*y*_*n*_) located at different distance classes (Dormann et al., 2007; Rangel et al., 2006). In the case of the SAR model, the space was considered by means of an additional parameter (ρ) that adjusted the relationship among neighboring sites (i.e., distances between them) by means of a spatial weights matrix (*W*), where the variance–covariance matrix takes the form: $C=\sigma^{2}{\left[{\left(I-\rho W\right)}^{T}\right]}^{-1}{\left[\left(I-\rho W\right)\right]}^{-1}$ (see Rangel et al., 2006). All models were constructed following Rangel, Field, and Diniz‐Filho (2011).

The spatially explicit models assume that the number of species in each location *i* has not only the function of an explicative variable (i.e., forest fragment area), but also the response values in nearby places *j* (richness in nearby fragments). Thus, the null hypothesis assumes that the size gradient of fragments does not affect the response to species richness when the geographic location of the fragments is considered (Dormann et al., 2007; Kissling & Carl, 2008; Quinn & Keough, 2002).

Previous to the analysis, the independent variable was transformed through the natural logarithm (Ln) given the difference in order of magnitude between the smallest (0.05 ha) and largest (936 ha) fragments. Similarly, the dependent variables (number of epiphytes and climbing plants) were transformed with the natural logarithm and the natural logarithm +1, respectively, since some fragments did not contain epiphytes. This transformation contributes to the normalization of residual errors and to the homogeneity of model variances (Quinn & Keough, 2002).

To select the model that best explained the pattern of species richness, we used the Akaike information criterion (AIC). This allows the regression models to be organized according to the smallest value of AIC, based on the principle of maximum likelihood (Burnham & Anderson, 2001; Johnson & Omland, 2004). It also reports on the contribution of the predictor ($R_{p}^{2}$) and the space ($R_{p+s}^{2}$) in the explanation of the model. We considered models with delta Akike values (the difference in AIC units from the highest‐ranking model) ≤2 to have strong support (Burnham & Anderson, 2001). All analyses were carried out using the SAM (Spatial Analysis in Macroecology) program V.4.0 (Rangel, Diniz‐Filho & Bini, 2010; Rangel et al., 2011).

### 2.4 Evaluation of the species composition nestedness

The nestedness of the species composition in the landscape was independently assessed for vascular epiphytes and climbing plants. We used both the presence–absence (0–1) and quantitative data sets. The quantitative data corresponded to the average abundance of the species per tree in a given fragment (e.g., number of fronds and stems per tree, respectively, observed in the total quadrants of a fragment; see Pincheira‐Ulbrich et al. (2016) and Supporting Information Appendix S1). Both types of matrices were constructed with the species in the rows and the forest fragments in the columns.

To perform the analyses, two matrices were constructed: the first matrix was ordered by columns according to the size gradient of the fragments, and the second matrix according to the isolation; then both were ordered by rows consistent with the occurrence or abundance of species (Atmar & Patterson, 1993; Lomolino, 1996; Ulrich et al., 2009). Thus, the force of the colonization and extinction processes in structuring the community was evaluated considering fragment size and isolation (Bruun & Moen, 2003; Cutler, 1994; Lomolino, 1996; Ulrich, 2009).

To estimate the degree of nestedness in the matrix, the NODF (nestedness measure based on overlap and decreasing fills) and WNODF (weighted nestedness metric based on overlap and decreasing fills) indexes were used. The use of both matrices permits the calculation of the contribution of the nestedness among sites or columns (i.e., species composition) or among species or rows (i.e., species incidence; Almeida‐Neto, Guimarães, Guimarães, Loyola, & Ulrich, 2008; Ulrich et al., 2009). The NODF index is calculated based on binary matrices (0–1), assuming that in a matrix with m rows (species) and n columns (site), row i is located above row j, and column k is located to the left of column l, according to the marginal total (i.e., the sum of 1's) of any column or row (e.g., Almeida‐Neto et al., 2008). The WNODF index is a modification of the former which allows for the use of quantitative data (i.e., species abundance). The indexes vary in scale from 0 to 100. Higher values indicate an increase in the degree of nestedness (Almeida‐Neto et al., 2008). The WNODF evaluates a pattern in which the subpopulations, which compose smaller local assemblages (fewer species), possess lower abundances than the populations which occur in the more abundant and richer assemblages (large fragments). Both indexes seem to be less sensitive to size and filling of the matrix than other commonly used matrices, and they are also less prone to type I error (Almeida‐Neto & Ulrich, 2011; Almeida‐Neto et al., 2008).

For the presence–absence data, the analysis considered a null model in which the marginal totals of the rows (species) remained fixed, while the marginal totals of the columns (fragments) were equiprobably randomly varied (Almeida‐Neto & Ulrich, 2011). This null model preserves the frequency of species occurrence and allows the species richness to vary equiprobably among forest fragments (e.g., Ulrich et al., 2009; Valencia‐Pacheco, Avaria‐Llautureo, Muñoz‐Escobar, Boric‐Bargetto, & Hernández, 2011). For the quantitative data, each matrix was randomly resampled keeping the presence–absence pattern fixed, allowing the species abundances to be assigned randomly (Ulrich, 2012). Finally, idiosyncratic species and sites—those that deviate from the general pattern nestedness—were determined. For this, the species and sites were randomized equiprobably from a uniform distribution (Ulrich, 2012). The statistical significance of the estimated nestedness indexes was obtained through the randomization of a null model with 10,000 simulations. The observed values were then compared with those estimated by the probability distribution of the null model considering intervals obtained at 95%. Any value obtained that varied within these limits was considered a random pattern (Almeida‐Neto & Ulrich, 2011). The matrix was prepared with the ECOSIM software (Gotelli & Entsminger, 2006) and the nestedness analyses were carried out with NODF software (Almeida‐Neto & Ulrich, 2011).

## 3 RESULTS

### 3.1 Evaluation of the species–area relationship

The adjustment of the SLM model (not including the effect of spatial autocorrelation) showed that a reduction in the size of forest fragments had a significant effect on the reduction in species richness of vascular epiphytes (SLM Model; *β* = 0.251, $R_{p}^{2}$ = 0.643; *p* < 0.001) and climbing plants (SLM model, *β* = 0.207; $R_{p}^{2}$ = 0.303; *p* < 0.001; Table 1). However, the AIC values showed that the best descriptive model of the species–area relationship was that which included the space autocorrelation effect, although the slope of the regression line (*β*) was statistically significant only for epiphytes (Table 1). In this last assemblage, the GLS (AIC = 44.917) and SAR (AIC = 45.178) models performed similarly (∆AICi = 0.261). However, considering that SAR requires an additional parameter, the GLS model seems more suitable (GLS, *β* = 0.33, $R_{p}^{2}$ = 0.579, *p* > 0.001). In climbing plants, the SAR model had a better performance, with a very small AIC (SAR, *β* = 0.136, $R_{p}^{2}$ = 0.268, *p* > 0.001, AIC = −18.498). These findings imply that a reduction in the size of forest fragments greatly decreases species richness in epiphyte assemblages (Table 1). The space autocorrelation effect, on the other hand, was more noticeable in climbing plants (SAR, $R_{p+s}^{2}$ = 0.912) than in epiphytes (GLS, $R_{p+s}^{2}$ = 0.714), indicating that the number of climbing species is more strongly influenced by the distance from other fragments (the neighborhood) than by fragment size (SAR Model, $R_{p}^{2}$ = 0.268).

**Table 1:** Regression models and their fit for the relationship among the area of 30 swamp forest fragments (*A*) and their species richness of climbing plants and vascular epiphytes

Parameters	Vascular epiphytes			Climbing plants		
	SLM	GLS	SAR	SLM	GLS	SAR
*c*	1.502	1.3	1.649	1.491	1.371	1.725
Β	0.251	0.33	0.207	0.108	0.177	0.136
p (c)	<0.001	<0.001	<0.001	<0.001	<0.001	<0.001
p (*β*)	***<0.001***	***<0.001***	***0.001***	***0.002***	***<0.001***	0.053
*ρ*			0.89			0.89
*EE* c	0.107	0.267	0.241	0.094	0.234	0.279
*EE β*	0.035	0.041	0.058	0.031	0.036	0.067
$R_{p}^{2}$	0.643	0.579	0.623	0.303	0.179	0.268
$R_{p+s}^{2}$		0.714	0.712		0.665	0.912
AIC	47.915	**44.917**	45.178	39.776	21.444	**−18.498**
∆AICi	2.998	0	0.261	58.274	39.942	0

Notes

Significant relationships are in bold and italics and the selected models are in bold.

$R_{p+s}^{2}$: coefficient of determination that considers the effect of the predictor plus the space; $R_{p}^{2}$: coefficient of determination that considers only the effect of the area (Ln(*A*)); ∆AICi: difference in AIC units from the highest‐ranking model; AIC: Akaike information criterion; EE: standard error of the estimation of the coefficients; GLS: generalized least squares model; p: probability associated with the results of the analysis of variance; SAR: spatial autoregressive model; SLM: simple linear regression model; *β*: slope of the regression line; *c*: intercept of the line with the *y*‐axis; *ρ*: autoregressive coefficient.

### 3.2 Evaluation of the species composition nestedness

The results of the nestedness analysis showed that the species (presence–absence) of vascular epiphytes and climbing plants are organized into a nested spatial pattern throughout the landscape, in relation to both the size gradient of swamp forest fragments and the distance from the largest fragment (NODF, *p* < 0.001; Table 2). Although the relationship with area was significant, nestedness could be considered medium–high, while it was higher in epiphytes (NODF = 65.95) than in climbing plants (NODF = 57.94). On the other hand, the results of nestedness in relation to distance from the largest fragment (isolation) were similar, but slightly less than those found for nestedness by area. Also, the effect of nestedness by isolation was higher in epiphytes (NODF = 54.17) than in climbers (NODF = 49.26). It is therefore important to point out that several unexpected absences and presences were found above and below the hypothetical diagonal that would exist under a perfectly nested pattern (Supporting Information Appendix S1). In both assemblages, the matrix rows contributed more to nesting than the columns, both in area and distance, although in both cases this contribution was markedly greater in epiphytes (NODFr = 89) than in climbers (NODFr = 70; Table 2). This indicates that local sets of species co‐occur along the forest fragment size gradient.

**Table 2:** Nestedness metrics for assemblages of vascular epiphyte species and climbing plants in 30 fragments of swamp forest

Assemblage	Data	Order	Metric	OV	SV	L95%	U95%	*p*(H0)
Vascular epiphytes	Presence–absence	Area	NODF	65.95	35.61	28.39	42.94	<0.001
			NODF_c_	59.77	28.76	19.71	37.96	<0.001
			NODF_r_	88.35	60.42	58.13	63.28	<0.001
		Distance	NODF	54.17	35.63	28.45	43.05	<0.001
			NODF_c_	44.74	28.79	19.73	38.00	<0.001
			NODFr	88.35	60.43	58.10	63.26	<0.001
	Quantitative (Average abundance per tree)	Area	WNODF	33	50.63	47.87	52.8	<0.001
			WNODF_c_	29.39	47.74	44.63	50.09	<0.001
			WNODF_r_	46.09	61.57	58.25	64.52	<0.001
		Distance	WNODF	27.33	41.08	39.01	42.87	<0.001
			WNODF_c_	22.16	35.56	33.44	37.31	<0.001
			WNODF_r_	46.09	61.56	58.23	64.52	<0.001
Climbing plants	Presence–absence	Area	NODF	57.94	36.28	28.30	44.61	<0.001
			NODF_c_	55.04	30.51	20.84	40.53	<0.001
			NODF_r_	69.97	60.18	55.56	65.27	<0.001
		Distance	NODF	49.26	36.28	28.21	44.49	0.001
			NODF_c_	44.26	30.50	20.65	40.39	0.003
			NODFr	69.97	60.23	55.68	65.53	0.001
	Quantitative (Average abundance per tree)	Area	WNODF	34.14	53.32	50.5	55.77	<0.001
			WNODF_c_	32.31	50.79	47.96	53.22	<0.001
			WNODF_r_	41.72	64.43	56.68	69.81	<0.001
		Distance	WNODF	28.75	41.59	39.24	43.55	<0.001
			WNODF_c_	25.63	36.21	33.97	38.1	<0.001
			WNODF_r_	41.72	64.45	56.58	69.71	<0.001

Note

NODF and WNODF = nestedness indexes for the complete matrix and for the contribution of rows (subscript r) and columns (subscript c). Order = rearrangement of the matrix by area or distance from the largest fragment. *p*(H0) = probability level of the null hypothesis of no difference between the observed (OV) and expected (SV) metrics, including the 95% confidence limits of SV (L95% – U95%) based on simulated values (10,000 iterations).

When considering the quantitative data (average abundance of species per tree), nestedness was significantly lower than both the results obtained with presence–absence data and significantly lower than those expected by chance (observed WNODF < simulated WNODF, *p* < 0.001, Table 2). This implies that the species abundance in the metacommunity is not nested, and in fact, an inverse pattern of abundance was found in relation to the size gradient and isolation of the fragments for both assemblages (quantitative data in Table 2).

This analysis also allowed for the detection of idiosyncratic species that deviated from the general nesting pattern, as was the case with the epiphytes *Asplenium trilobum* Cav. and *Polypodium feuillei* Bertero, and the climbers *Cissus striata* Ruiz & Pav. and *Ercilla spp*. The observed atypical pattern may be the result of the differentiated effect of habitat fragmentation on the abundance of these species and the effect of rare species, only observed in some sites. This uncharacteristic pattern also occurred in idiosyncratic sites, as it did for epiphytes in the 55 ha fragment, since, even though it is far from the largest fragment, its own size proved sufficient to maintain many abundant species. In the case of climbers, a greater number of idiosyncratic sites and species were found in terms of both area and distance, which may indicate a lesser effect of habitat fragmentation (Supporting Information Appendix S1).

## 4 DISCUSSION

In this study, we found that a reduction in the size of forest fragments significantly decreased the species richness of vascular epiphytes (*β* = 0.33, *p* < 0.001), but not of climbing plants (*β* = 0.136, *p* = 0.053). These results indicate that community dynamics are driven, to some extent, by the size gradient of the fragments (area per se hypothesis). Consequently, the size of forest fragments is a better predictor of epiphytes richness, while the distance from the largest fragment or the amount of neighboring fragments plays a greater role for the community of climbing plants.

The underlying explanations for these findings can be attributed to epiphytes’ strong dependence on trees (Bartels & Chen, 2012) and the dispersion of seeds with protected embryos (Arroyo‐Rodríguez & Toledo‐Acevedo, 2009; Campbell et al., 2015; Mohandass et al., 2014). In these temperate South American forests, seeds are dispersed mainly by birds (see Armesto & Rozzi, 1989) and germinate with different success rates in both open and closed canopy sites (Figueroa, 2003), with a maximum dispersion distance of 102 m (Núñes‐Avila, Uriarte, Marquet, & Armesto, 2013). On the other hand, the epiphytes studied here were mainly filmy ferns (i.e., Hymenophyllaceae), with delicate bodies and spores dispersed (potentially several kilometers) by the wind, that develop on the bark of trees and greatly depend upon the humidity of microsites to germinate (Muñoz et al., 2003; Parra et al., 2009; San Martín et al., 2008; Woda et al., 2006; Zotz, 2016). The Hymenophyllaceae family has green spores (chlorophyll), which are presumably more vulnerable to extreme weather conditions (drought or cold) and have a short life span (48 days on average) compared to spores without chloroplast, which can last for months or years. Green spores can nonetheless live long enough to colonize a wide range of distribution (Mehltreter 2010). Climbers were consequently found in all sites, while epiphytes were typically not found in small fragments exposed to the landscape matrix (i.e., 0.7, 0.12, and 0.07 ha). This indicates that epiphytes could be more severely affected by local processes (i.e., changes in forest structure) than climbers. Thus, dispersal in a fragmented neighborhood seems to be less important in determining epiphyte richness than climbers (see Larrea & Werner, 2010; Pereira & Cavalcanti, 2007).

The species–area relationship is a well‐described pattern for many vascular plant assemblages (e.g., Cagnolo, Cabido, & Valladares, 2006; Echeverría et al., 2007; Pincheira‐Ulbrich et al., 2009), and the evidence supports the area per se hypothesis as one of the key determinants of vascular epiphyte communities in fragmented landscapes (Köster, Friedrich, Nieder, & Barthlott, 2009; Pincheira‐Ulbrich et al., 2012). This is in accordance with previous studies which sustain that fragmentation and habitat loss are the most important causes of global biodiversity loss (Fahrig, 2003; Haddad et al., 2015; Tilman et al., 1994). However, some organisms do not conform as clearly to this pattern, as in the case of climbing plants, because they seem to benefit (up to a certain level) from the fragmentation process (e.g., Schnitzer & Bongers, 2002). These findings are particularly reliable because we have used inventories whose completeness has been evaluated previously (Pincheira‐Ulbrich et al., 2016), and the space has been controlled to avoid spurious results (see Domínguez et al., 2012; Hortal et al., 2007; Rivera‐Huntiel et al., 2012).

The loss of area, apart from its consequences for the number of species, also had a significant effect on the nestedness pattern of the metacommunity species composition. This result is to be expected because the nestedness pattern has proven seems to be common in fragmented landscapes, such as islands, mountain peaks and forest patches (e.g., Bruun & Moen, 2003; Honnay, Hermy, & Coppin, 1999; Wright, Patterson, Mikkelson, Cutler, & Atmar, 1998). This gradual change in the species composition may be the result of the change in the assembly of species from less to more sensitive to the core‐edge relationship, in fragments that gradually become smaller and more influenced by the landscape matrix (e.g., Honnay et al. 1999). Although it could be assumed that a habitat's structure is the only factor necessary to explain changes in the species assembly, the theories of biogeography of islands and metacommunities assume the connection of habitats through dispersion, therefore, local and metacommunity processes are continuously operating, and consequently, the effect of space is assumed in both theories (Leibold et al., 2004; Macarthur & Wilson, 2001).

Nestedness was also observed when the matrix was arranged in relation to by distance from to the largest fragment, although the effect was smaller than that produced by area. These findings (i.e., the area and isolation effect) indicate that there is a greater turnover of climbing plants than epiphytic species in the landscape (beta diversity), a pattern that has been described in previous studies for the first of these functional groups (e.g., Burnham, 2004). Here, the local colonization of climbing plants was found to be mediated principally by the distance among fragments. On the other hand, in the epiphyte assemblages selective extinction was mediated predominantly by fragment area size (see Patterson & Atmar, 1986; Ulrich, 2012; Ulrich et al., 2009), a pattern that could be explained by the loss of local microhabitats associated with the decrease in fragment size. Consequently, the structuring of assemblages throughout the landscape appears to be influenced by a source–sink dynamic, which would affect epiphytes more than climbing plants. In this way, small forest fragments could be considered to constitute marginal environments maintained by the arrival of propagules (sink habitats) from larger, better‐conserved fragments (source habitats; e.g., Leibold et al., 2004).

Accordingly, the finding of a larger amount of idiosyncratic species in climbing plant assemblages is consistent with the hypothesis of selective colonization (e.g., Patterson, 1990). Thus, the nestedness of climbers is organized in a less orderly sequence, while epiphytes tend to show a more defined pattern throughout the landscape as a response to the fragment's area and the distance from a large fragment—the hypothetical source of colonization (See Atmar & Patterson, 1993; Lomolino, 1996; Ulrich et al., 2009). However, in both assemblages we found that the contribution of rows to nestedness was greater than that of columns, indicating that the co‐occurrence of local sets of species is first associated with similar ecological requirements among species and then with environmental gradients that could vary with fragment size.

On the other hand, the WNODF was inversely related to the size gradient of the fragments and to their isolation, suggesting that as fragment size diminishes they gradually sustain fewer species, but these particular species would be more abundant than in larger fragments. This indicates that the abundances are not nested and could depend on local scale microhabitat variability (Mehltreter 2010, Zotz, 2016) and stochasticity (Shaffer, 1981). This pattern could represent the result of the synergy between local competition and habitat heterogeneity (Leibold et al., 2004; Tilman et al., 1994), which may directly affect abundance, but not necessarily the occurrence of species. Further studies are nonetheless required to evaluate these hypotheses, such as, the experimental evaluation of the competition among species for different substrate types, or exclusion studies of one or more species in a single type of substrate to evaluate the changes of abundance in time and space (Logue, Mouquet, Peter, & Hillebrand, 2011).

In metacommunities, the presence of strong nestedness is a clear indication of coupled gradients of site environmental characteristics and species traits (Ulrich et al., 2009). It could thus be inferred that as fragments become smaller, specialist species are gradually replaced by general habitat species (Cutler, 1994; Echeverría et al., 2007; Pereira & Cavalcanti, 2007; Ulrich et al., 2009). For example, the presence of *Grammitis magellanica* could indicate a less altered habitat because its green spores are presumably highly vulnerable to extreme microclimatic conditions (drought or cold) and have a short life span. (Mehltreter 2010), which is the potential reason why *G. magellanica* was scarcely found in medium and small fragments (see Supporting Information Appendix S1). This leads to the assumption that environmental conditions change gradually with fragment size (Leibold et al., 2004; Massol et al., 2011). The environment of the patch can thus act as a filter, ordering species composition within its area (Leibold et al., 2004). The latter is especially the case in edge habitats, which constitute areas that limit the development of a wide variety of species (e.g., filmy ferns) that are less tolerant to these microclimatic conditions (e.g., higher solar radiation and less moisture), and at the same time extend the habitat of other species that prefer these areas (e.g., climbing plants; Fahrig, 2003; Gascon et al., 2000; López‐Barrera et al., 2007; Murcia, 1995).

The literature discusses additional factors that may influence nesting of species, such as passive sampling, habitat nesting and habitat heterogeneity (Ulrich et al., 2009). In passive sampling, abundant species have a better chance of colonizing many patches than low‐density species. For example, the epiphytic fern *Hymenophyllum plicatum* or the climber *Cissus striata* are abundant not only in these forests, but throughout the whole temperate forest region, and were therefore present in most of the sites sampled. In epiphytes, habitat nesting may occur due to the presence of large trees and decaying logs left in different sites as remnants of anthropic activity, which could provide propagules that maintain local diversity (see Cutler, 1994; Pincheira‐Ulbrich et al., 2012; Ulrich et al., 2009). In the case of climbing plants, their variation throughout the landscape has also been found to be influenced by the presence of canopy gaps (e.g., Ibarra‐Manriquez & Martínez‐Ramos, 2002; Malizia & Grau, 2008; Schnitzer & Bongers, 2002). Thus, different assemblages may respond differentially to the size and quality of the fragment, which is associated with different rates of extinction and colonization according to the particular life history of the species (Cagnolo, Valladares, Salvo, Cabido, & Zak, 2009; Collins, Holt, & Foster, 2009; Ewers & Didham, 2005; Miller, Quintana‐Ascencio, Maliakal‐Witt, & Menges, 2011; Prevedello & Vieira, 2010; Saldaña, Parra, Flores‐Bavestrello, Corcuera, & Bravo, 2014; Woda et al., 2006), as evidenced in our study.

Finally, the fragment size (e.g., for epiphytes) and isolation (e.g., for climbing plants) are likely the most important variables for the conservation of these assemblages, since large fragments maintain more species and have lower extinction rates (Cutler, 1994; Echeverría et al., 2007; Ulrich et al., 2009), while proximity favors dispersal among forest fragments (Watson et al., 2004). For example, the metacommunity may be affected by local extinctions when the extinction rate is higher than the immigration rate from other patches. Near the threshold of extinction, most of the fragments will be small and will almost inevitably contain relatively small populations of most species (Campbell et al., 2015; Haddad et al., 2015; Larrea & Werner, 2010; Pincheira‐Ulbrich et al., 2016; Zotz & Bader, 2009).

According to Shaffer (1981), the underlying mechanisms that lead to this threshold can be attributed to demographic stochasticity (e.g., reproductive success), environmental stochasticity (e.g., changes in luminosity levels in the habitat), natural disasters (e.g., forest fires), and the reduction of genetic diversity (e.g., changes in allelic frequencies). Thus, the anthropogenic matrix and the fragmentation process impose restrictions on the dispersal and establishment of epiphytes (Larrea & Werner, 2010), increasing the probability of local extinction, while climbing plants have proven to be less affected by habitat anthropization and reduction (e.g., Arroyo‐Rodríguez & Toledo‐Acevedo, 2009; Campbell et al., 2015; Mohandass et al., 2014). Therefore, the explicit inclusion in future research of the specific human activities creating the fragmentation seems necessary. Nonetheless, the nestedness of a metacommunity provides it with high resilience to recover from historical fragmentation and disturbance, mainly because the system becomes more redundant, increasing resistance and resilience to disturbances (Cook & Quinn, 1995; Pagel, Martínez‐Abraín, Gómez, Jiménez, & Oro, 2014).

## 5 CONCLUSIONS

Our evaluation of the species–area relationship and nestedness of species composition of vascular epiphytes and climbing plants in fragments of swamp forest supports the hypothesis that a reduction in the area of fragments has profound consequences by reducing the species number in epiphytes. While this effect was marginal in climbing plants, the control of the geographic space in this evaluation enabled us to clearly show that the richness of climbers depended strongly on belonging to a neighborhood of fragments. A general pattern of nestedness of the matrix for both plant assemblages was found; in accordance with previous studies, though this was higher in epiphytes, which can be attributed to the source–sink dynamics of the metacommunity. Consequently, local colonization processes mediated by isolation proved to be the predominant mechanisms determining the spatial configuration of the climbing plant metacommunity, while selective extinction mediated by area size characterized the epiphyte metacommunity.

## CONFLICT OF INTEREST

None declared.

## AUTHOR CONTRIBUTIONS

J.P.U. and C.E.H. conceived of the ideas; J.P.U. collected the data; J.P.U, C.E.H. and A.S. analyzed the data; J.P.U wrote the first manuscript draft. All of the authors contributed considerably to the data interpretation and the preparation of the final manuscript.

## DATA ACCESSIBILITY

All data supporting this study are provided as appendix information accompanying this paper. In addition, the data may be uploaded to Dryad or another database with similar characteristics. https://doi.org/10.5061/dryad.p546pb2.
