# Supplementary material for: Consequences of swamp forest fragmentation on assemblages of vascular epiphytes and climbing plants: Evaluation of the metacommunity structure
Source: Ecol Evol. 2018 Nov 14;8(23):11785–98. doi: 10.1002/ece3.4635 (PMC6303743; doi:10.1002/ece3.4635)
Supplement: Supplementary file 1 [file ECE3-8-11785-s001.docx]

Appendix

Nestedness matrices of species composition (in rows) of vascular epiphytes (n = 16 species) and climbing plants (n = 15 species) in 30 fragments of swamp forest (in columns). Order of columns: area = columns sorted by size of the fragments (ha), distance = columns sorted by distance to the largest fragment (in brackets distance in km to the largest fragment). Type of data: presence-absence = matrix based on 0 and 1, Quantitative = matrix based on the average abundance of the species per tree (e.g. number of fronds in epiphytes and number of stems in climbers). All Matrices were secondarily ordered by the sum of marginal totals (species). * = idiosyncratic species or sites. Note: most of the epiphytic species were ferns whit the exception of *Sarmienta scandens* (Fam: Gesneriaceae) and *Fascicularia bicolor* (Fam: Bromeliceae).

1. Presence-absence matrix of epiphytes ordered by area.

| Specie | 936 | 156 | 133 | 86 | 67 | 55 | 50 | 42 | 40 | 33 | 22 | 16 | 11 | 9.4 | 7.1 | 5.5 | 3.9 | 3.4 | 3 | 1.9 | 1.8 | 1 | 0.8 | 0.7 | 0.15 | 0.14 | 0.13 | 0.12 | 0.07 | 0.05 | Total |
| --- | --- | --- | --- | --- | --- | --- | --- | --- | --- | --- | --- | --- | --- | --- | --- | --- | --- | --- | --- | --- | --- | --- | --- | --- | --- | --- | --- | --- | --- | --- | --- |
| *Hymenophyllum plicatum* | 1 | 1 | 1 | 1 | 1 | 1 | 1 | 1 | 1 | 1 | 1 | 1 | 1 | 1 | 1 | 1 | 1 | 1 | 1 | 1 | 1 | 1 | 0 | 0 | 1 | 1 | 0 | 0 | 0 | 1 | 25 |
| *Asplenium trilobum** | 1 | 1 | 1 | 1 | 0 | 0 | 1 | 1 | 1 | 1 | 1 | 1 | 1 | 1 | 1 | 0 | 1 | 1 | 1 | 1 | 1 | 1 | 1 | 0 | 1 | 1 | 1 | 0 | 0 | 1 | 23 |
| *Aspleium dareoides* | 1 | 1 | 1 | 1 | 1 | 1 | 1 | 1 | 1 | 1 | 1 | 1 | 1 | 1 | 1 | 1 | 0 | 1 | 1 | 1 | 1 | 1 | 0 | 0 | 0 | 0 | 0 | 0 | 0 | 0 | 21 |
| *Sarmienta scandens* | 1 | 1 | 1 | 1 | 1 | 1 | 1 | 1 | 1 | 1 | 1 | 1 | 1 | 1 | 1 | 1 | 0 | 1 | 1 | 1 | 1 | 1 | 0 | 0 | 0 | 0 | 0 | 0 | 0 | 1 | 21 |
| *Polypodium feuyllei** | 1 | 1 | 1 | 0 | 1 | 1 | 1 | 1 | 1 | 1 | 0 | 1 | 1 | 0 | 1 | 1 | 1 | 0 | 1 | 1 | 1 | 1 | 1 | 0 | 1 | 1 | 1 | 0 | 0 | 1 | 20 |
| *Hymenophyllum krauseanum* | 1 | 1 | 1 | 1 | 1 | 1 | 1 | 1 | 1 | 1 | 1 | 1 | 1 | 1 | 1 | 1 | 0 | 1 | 0 | 0 | 1 | 1 | 0 | 0 | 0 | 0 | 0 | 0 | 0 | 0 | 19 |
| *Fascicularia bicolor* | 1 | 1 | 1 | 1 | 1 | 1 | 1 | 0 | 1 | 1 | 1 | 1 | 1 | 1 | 1 | 1 | 0 | 1 | 0 | 0 | 0 | 1 | 0 | 0 | 0 | 0 | 0 | 0 | 0 | 0 | 16 |
| *Hymenophyllum caudiculatum* | 1 | 1 | 1 | 1 | 1 | 1 | 1 | 0 | 1 | 1 | 1 | 0 | 0 | 1 | 1 | 1 | 0 | 1 | 0 | 1 | 0 | 1 | 0 | 0 | 0 | 0 | 0 | 0 | 0 | 0 | 15 |
| *Hymenophyllum dentatum* | 1 | 1 | 1 | 1 | 1 | 1 | 1 | 0 | 1 | 1 | 1 | 1 | 0 | 1 | 1 | 0 | 0 | 1 | 0 | 0 | 0 | 0 | 0 | 0 | 0 | 0 | 0 | 0 | 0 | 0 | 14 |
| *Hymenophyllum peltatum* | 1 | 1 | 1 | 0 | 1 | 1 | 1 | 0 | 1 | 1 | 1 | 0 | 0 | 1 | 0 | 1 | 0 | 0 | 0 | 0 | 0 | 0 | 0 | 0 | 0 | 0 | 0 | 0 | 0 | 0 | 11 |
| *Hymenophyllum dicranotrichum* | 1 | 1 | 1 | 1 | 1 | 1 | 1 | 0 | 0 | 1 | 0 | 0 | 0 | 0 | 0 | 1 | 0 | 0 | 0 | 1 | 0 | 0 | 0 | 0 | 0 | 0 | 0 | 0 | 0 | 0 | 10 |
| *Hymenoglossum cruentum* | 1 | 1 | 1 | 1 | 1 | 1 | 1 | 0 | 0 | 1 | 0 | 0 | 0 | 0 | 0 | 0 | 0 | 0 | 0 | 0 | 0 | 0 | 0 | 0 | 0 | 0 | 0 | 0 | 0 | 0 | 8 |
| *Hymenophyllum cuneatum* | 1 | 1 | 1 | 1 | 0 | 0 | 1 | 0 | 1 | 1 | 1 | 0 | 0 | 0 | 0 | 0 | 0 | 0 | 0 | 0 | 0 | 0 | 0 | 0 | 0 | 0 | 0 | 0 | 0 | 0 | 7 |
| *Hymenophyllum pectinatum* | 1 | 1 | 1 | 1 | 1 | 1 | 1 | 0 | 0 | 0 | 0 | 0 | 0 | 1 | 0 | 0 | 0 | 0 | 0 | 0 | 0 | 0 | 0 | 0 | 0 | 0 | 0 | 0 | 0 | 0 | 7 |
| *Grammitis magellanica* | 1 | 1 | 1 | 0 | 0 | 0 | 0 | 0 | 1 | 1 | 0 | 0 | 0 | 0 | 1 | 0 | 0 | 0 | 0 | 0 | 0 | 0 | 0 | 0 | 0 | 0 | 0 | 0 | 0 | 0 | 6 |
| *Hymenophyllum secundum* | 1 | 1 | 1 | 0 | 0 | 0 | 0 | 0 | 0 | 0 | 0 | 0 | 0 | 0 | 0 | 0 | 0 | 0 | 0 | 0 | 0 | 0 | 0 | 0 | 0 | 0 | 0 | 0 | 0 | 0 | 3 |
| Total | 16 | 16 | 14 | 10 | 11 | 11 | 14 | 6 | 12 | 12 | 10 | 8 | 7 | 10 | 10 | 9 | 3 | 8 | 4 | 7 | 6 | 8 | 2 | 0 | 3 | 3 | 2 | 0 | 0 | 4 |  |

1. Presence-absence matrix of epiphytes ordered by distance.

| Specie | 936  (0) | 9.4  (8) | 0.8  (11) | 40 (15) | 133 (15.2) | 67 (15.7) | 33  (15.8) | 22  (17) | 5.5 (17.3) | 7.1 (18) | 0.15 (21) | 3.4 (21,5) | 156 (21.6) | 86 (22.6) | 1  (24) | 0.07 (26.5) | 0.12 (26.8) | 0.13 (27) | 3.9 (27.5) | 1.9 (28.3) | 0.14 (28.4) | 0.05 (29) | 55 (30) | 50 (32) | 11 (40) | 16 (40.1) | 0.7 (40.5) | 3 (42) | 1.8 (47) | 42 (60) | Total |
| --- | --- | --- | --- | --- | --- | --- | --- | --- | --- | --- | --- | --- | --- | --- | --- | --- | --- | --- | --- | --- | --- | --- | --- | --- | --- | --- | --- | --- | --- | --- | --- |
| *Hymenophyllum plicatum* | 1 | 1 | 0 | 1 | 1 | 1 | 1 | 1 | 1 | 1 | 1 | 1 | 1 | 1 | 1 | 0 | 0 | 0 | 1 | 1 | 1 | 1 | 1 | 1 | 1 | 1 | 0 | 1 | 1 | 1 | 25 |
| *Asplenium trilobum* | 1 | 1 | 1 | 1 | 1 | 0 | 1 | 1 | 0 | 1 | 1 | 1 | 1 | 1 | 1 | 0 | 0 | 1 | 1 | 1 | 1 | 1 | 0 | 1 | 1 | 1 | 0 | 1 | 1 | 1 | 23 |
| *Aspleium dareoides* | 1 | 1 | 0 | 1 | 1 | 1 | 1 | 1 | 1 | 1 | 0 | 1 | 1 | 1 | 1 | 0 | 0 | 0 | 0 | 1 | 0 | 0 | 1 | 1 | 1 | 1 | 0 | 1 | 1 | 1 | 21 |
| *Sarmienta scandens* | 1 | 1 | 0 | 1 | 1 | 1 | 1 | 1 | 1 | 1 | 0 | 1 | 1 | 1 | 1 | 0 | 0 | 0 | 0 | 1 | 0 | 1 | 1 | 1 | 1 | 1 | 0 | 1 | 1 | 1 | 21 |
| *Polypodium feuyllei** | 1 | 0 | 1 | 1 | 1 | 1 | 1 | 0 | 1 | 1 | 1 | 0 | 1 | 0 | 1 | 0 | 0 | 1 | 1 | 1 | 1 | 1 | 1 | 1 | 1 | 1 | 0 | 1 | 1 | 1 | 20 |
| *Hymenophyllum krauseanum* | 1 | 1 | 0 | 1 | 1 | 1 | 1 | 1 | 1 | 1 | 0 | 1 | 1 | 1 | 1 | 0 | 0 | 0 | 0 | 0 | 0 | 0 | 1 | 1 | 1 | 1 | 0 | 0 | 1 | 1 | 19 |
| *Fascicularia bicolor* | 1 | 1 | 0 | 1 | 1 | 1 | 1 | 1 | 1 | 1 | 0 | 1 | 1 | 1 | 1 | 0 | 0 | 0 | 0 | 0 | 0 | 0 | 1 | 1 | 1 | 1 | 0 | 0 | 0 | 0 | 16 |
| *Hymenophyllum caudiculatum* | 1 | 1 | 0 | 1 | 1 | 1 | 1 | 1 | 1 | 1 | 0 | 1 | 1 | 1 | 1 | 0 | 0 | 0 | 0 | 1 | 0 | 0 | 1 | 1 | 0 | 0 | 0 | 0 | 0 | 0 | 15 |
| *Hymenophyllum dentatum* | 1 | 1 | 0 | 1 | 1 | 1 | 1 | 1 | 0 | 1 | 0 | 1 | 1 | 1 | 0 | 0 | 0 | 0 | 0 | 0 | 0 | 0 | 1 | 1 | 0 | 1 | 0 | 0 | 0 | 0 | 14 |
| *Hymenophyllum peltatum* | 1 | 1 | 0 | 1 | 1 | 1 | 1 | 1 | 1 | 0 | 0 | 0 | 1 | 0 | 0 | 0 | 0 | 0 | 0 | 0 | 0 | 0 | 1 | 1 | 0 | 0 | 0 | 0 | 0 | 0 | 11 |
| *Hymenophyllum dicranotrichum* | 1 | 0 | 0 | 0 | 1 | 1 | 1 | 0 | 1 | 0 | 0 | 0 | 1 | 1 | 0 | 0 | 0 | 0 | 0 | 1 | 0 | 0 | 1 | 1 | 0 | 0 | 0 | 0 | 0 | 0 | 10 |
| *Hymenoglossum cruentum* | 1 | 0 | 0 | 0 | 1 | 1 | 1 | 0 | 0 | 0 | 0 | 0 | 1 | 1 | 0 | 0 | 0 | 0 | 0 | 0 | 0 | 0 | 1 | 1 | 0 | 0 | 0 | 0 | 0 | 0 | 8 |
| *Hymenophyllum cuneatum* | 1 | 0 | 0 | 1 | 1 | 0 | 1 | 1 | 0 | 0 | 0 | 0 | 1 | 1 | 0 | 0 | 0 | 0 | 0 | 0 | 0 | 0 | 0 | 1 | 0 | 0 | 0 | 0 | 0 | 0 | 7 |
| *Hymenophyllum pectinatum* | 1 | 1 | 0 | 0 | 1 | 1 | 0 | 0 | 0 | 0 | 0 | 0 | 1 | 1 | 0 | 0 | 0 | 0 | 0 | 0 | 0 | 0 | 1 | 1 | 0 | 0 | 0 | 0 | 0 | 0 | 7 |
| *Grammitis magellanica* | 1 | 0 | 0 | 1 | 1 | 0 | 1 | 0 | 0 | 1 | 0 | 0 | 1 | 0 | 0 | 0 | 0 | 0 | 0 | 0 | 0 | 0 | 0 | 0 | 0 | 0 | 0 | 0 | 0 | 0 | 6 |
| *Hymenophyllum secundum* | 1 | 0 | 0 | 0 | 1 | 0 | 0 | 0 | 0 | 0 | 0 | 0 | 1 | 0 | 0 | 0 | 0 | 0 | 0 | 0 | 0 | 0 | 0 | 0 | 0 | 0 | 0 | 0 | 0 | 0 | 3 |
| Total | 16 | 10 | 2 | 12 | 14 | 11 | 12 | 10 | 9 | 10 | 3 | 8 | 16 | 10 | 8 | 0 | 0 | 2 | 3 | 7 | 3 | 4 | 11 | 14 | 7 | 8 | 0 | 4 | 6 | 6 |  |

1. Quantitative matrix of epiphytes ordered by area.

| Especie | 936 | 156 | 133 | 86 | 67 | 55 | 50 | 42 | 40 | 33 | 22 | 16 | 11 | 9.4 | 7.1 | 5.5 | 3.9 | 3.4 | 3 | 1.9 | 1.8 | 1 | 0.8 | 0.7 | 0.15 | 0.14 | 0.13 | 0.12 | 0.07 | 0.05 | Total |
| --- | --- | --- | --- | --- | --- | --- | --- | --- | --- | --- | --- | --- | --- | --- | --- | --- | --- | --- | --- | --- | --- | --- | --- | --- | --- | --- | --- | --- | --- | --- | --- |
| *Hymenophyllum plicatum* | 16.6 | 6.8 | 41.6 | 17.2 | 18.3 | 6.1 | 13.0 | 0.2 | 3.6 | 53.4 | 6.3 | 1.7 | 17.7 | 29.9 | 15.6 | 1.9 | 28.7 | 5.2 | 1.2 | 65.0 | 3.0 | 2.1 | 0.0 | 0.0 | 1.3 | 55.8 | 0.0 | 0.0 | 0.0 | 2.4 | 415 |
| *Hymenophyllum caudiculatum* | 0.3 | 19.9 | 6.9 | 87.2 | 17.3 | 36.7 | 37.8 | 0.0 | 2.3 | 0.0 | 17.6 | 0.0 | 0.0 | 2.9 | 0.5 | 1.0 | 0.0 | 1.5 | 0.0 | 3.3 | 0.0 | 7.7 | 0.0 | 0.0 | 0.0 | 0.0 | 0.0 | 0.0 | 0.0 | 0.0 | 243 |
| *Aspleium dareoides* | 6.6 | 2.6 | 21.1 | 5.4 | 5.1 | 1.6 | 4.6 | 8.8 | 0.7 | 23.9 | 4.9 | 0.4 | 0.7 | 28.7 | 12.3 | 12.2 | 0.0 | 2.3 | 0.7 | 0.6 | 3.1 | 4.4 | 0.0 | 0.0 | 0.0 | 0.0 | 0.0 | 0.0 | 0.0 | 0.0 | 151 |
| *Hymenophyllum dicranotrichum* | 9.5 | 10.2 | 0.9 | 47.1 | 15.4 | 12.5 | 43.0 | 0.0 | 0.0 | 2.1 | 0.0 | 0.0 | 0.0 | 0.0 | 0.0 | 1.7 | 0.0 | 0.0 | 0.0 | 2.9 | 0.0 | 0.0 | 0.0 | 0.0 | 0.0 | 0.0 | 0.0 | 0.0 | 0.0 | 0.0 | 145 |
| *Asplenium trilobum** | 1.8 | 0.9 | 3.4 | 12.9 | 0.0 | 0.0 | 0.2 | 1.9 | 0.2 | 0.0 | 0.2 | 0.1 | 1.7 | 12.8 | 7.3 | 0.0 | 7.4 | 3.0 | 4.7 | 8.6 | 4.7 | 34.2 | 0.1 | 0.0 | 0.8 | 9.2 | 22.3 | 0.0 | 0.0 | 3.2 | 142 |
| *Hymenophyllum krauseanum* | 10.1 | 12.1 | 33.5 | 0.4 | 10.7 | 5.3 | 4.1 | 0.3 | 0.5 | 7.9 | 0.2 | 0.1 | 12.5 | 2.7 | 4.8 | 5.1 | 0.0 | 1.4 | 0.0 | 0.0 | 1.0 | 7.2 | 0.0 | 0.0 | 0.0 | 0.0 | 0.0 | 0.0 | 0.0 | 0.0 | 120 |
| *Hymenoglossum cruentum* | 0.1 | 2.7 | 3.1 | 20.4 | 5.4 | 5.4 | 3.4 | 0.0 | 0.0 | 7.4 | 0.0 | 0.0 | 0.0 | 0.0 | 0.0 | 0.0 | 0.0 | 0.0 | 0.0 | 0.0 | 0.0 | 0.0 | 0.0 | 0.0 | 0.0 | 0.0 | 0.0 | 0.0 | 0.0 | 0.0 | 48 |
| *Hymenophyllum peltatum* | 8.3 | 5.7 | 4.0 | 0.0 | 2.8 | 3.6 | 4.6 | 0.0 | 0.0 | 5.0 | 0.9 | 0.0 | 0.0 | 1.1 | 0.0 | 0.3 | 0.0 | 0.0 | 0.0 | 0.0 | 0.0 | 0.0 | 0.0 | 0.0 | 0.0 | 0.0 | 0.0 | 0.0 | 0.0 | 0.0 | 36 |
| *Sarmienta scandens* | 1.6 | 1.8 | 0.9 | 0.2 | 4.6 | 0.9 | 0.8 | 0.7 | 0.1 | 0.3 | 0.5 | 0.5 | 2.6 | 0.3 | 1.5 | 0.2 | 0.0 | 0.1 | 0.9 | 10.3 | 0.4 | 0.5 | 0.0 | 0.0 | 0.0 | 0.0 | 0.0 | 0.0 | 0.0 | 1.9 | 32 |
| *Grammitis magellanica* | 0.1 | 0.1 | 4.8 | 0.0 | 0.0 | 0.0 | 0.0 | 0.0 | 0.2 | 22.3 | 0.0 | 0.0 | 0.0 | 0.0 | 0.2 | 0.0 | 0.0 | 0.0 | 0.0 | 0.0 | 0.0 | 0.0 | 0.0 | 0.0 | 0.0 | 0.0 | 0.0 | 0.0 | 0.0 | 0.0 | 28 |
| *Fascicularia bicolor* | 0.3 | 0.2 | 3.9 | 0.9 | 1.1 | 2.7 | 0.5 | 0.0 | 0.1 | 0.5 | 0.9 | 0.1 | 0.2 | 0.2 | 0.5 | 0.1 | 0.0 | 0.3 | 0.0 | 0.0 | 0.0 | 0.7 | 0.0 | 0.0 | 0.0 | 0.0 | 0.0 | 0.0 | 0.0 | 0.0 | 13 |
| *Polypodium feuyllei** | 0.2 | 0.0 | 0.2 | 0.0 | 0.1 | 0.1 | 0.0 | 0.5 | 0.0 | 0.1 | 0.0 | 0.4 | 0.1 | 0.0 | 0.1 | 0.2 | 1.1 | 0.0 | 0.0 | 0.1 | 0.5 | 0.2 | 0.1 | 0.0 | 0.5 | 4.1 | 2.8 | 0.0 | 0.6 | 0.4 | 12 |
| *Hymenophyllum dentatum* | 1.6 | 0.9 | 0.3 | 0.1 | 4.6 | 0.1 | 1.0 | 0.0 | 0.1 | 1.7 | 0.2 | 0.0 | 0.0 | 0.1 | 0.5 | 0.0 | 0.0 | 0.6 | 0.0 | 0.0 | 0.0 | 0.0 | 0.0 | 0.0 | 0.0 | 0.0 | 0.0 | 0.0 | 0.0 | 0.0 | 12 |
| *Hymenophyllum cuneatum* | 0.0 | 1.9 | 0.3 | 0.4 | 0.0 | 0.0 | 5.4 | 0.0 | 0.8 | 0.2 | 0.0 | 0.0 | 0.0 | 0.0 | 0.0 | 0.0 | 0.0 | 0.0 | 0.0 | 0.0 | 0.0 | 0.0 | 0.0 | 0.0 | 0.0 | 0.0 | 0.0 | 0.0 | 0.0 | 0.0 | 9 |
| *Hymenophyllum pectinatum* | 0.1 | 0.6 | 0.1 | 3.7 | 0.7 | 0.2 | 1.8 | 0.0 | 0.0 | 0.0 | 0.0 | 0.0 | 0.0 | 1.1 | 0.0 | 0.0 | 0.0 | 0.0 | 0.0 | 0.0 | 0.0 | 0.0 | 0.0 | 0.0 | 0.0 | 0.0 | 0.0 | 0.0 | 0.0 | 0.0 | 8 |
| *Hymenophyllum secundum* | 0.1 | 1.0 | 0.4 | 0.0 | 0.0 | 0.0 | 0.0 | 0.0 | 0.0 | 0.0 | 0.0 | 0.0 | 0.0 | 0.0 | 0.0 | 0.0 | 0.0 | 0.0 | 0.0 | 0.0 | 0.0 | 0.0 | 0.0 | 0.0 | 0.0 | 0.0 | 0.0 | 0.0 | 0.0 | 0.0 | 1 |
| Total | 57 | 67 | 126 | 196 | 86 | 75 | 120 | 12 | 9 | 125 | 32 | 3 | 35 | 80 | 43 | 23 | 37 | 15 | 8 | 91 | 13 | 57 | 0 | 0 | 3 | 69 | 25 | 0 | 1 | 8 |  |

1. Quantitative matrix of epiphytes ordered by distance.

| Especie | 936  (0) | 9.4  (8) | 0.8  (11) | 40 (15) | 133 (15.2) | 67 (15.7) | 33  (15.8) | 22  (17) | 5.5 (17.3) | 7.1 (18) | 0.15 (21) | 3.4 (21,5) | 156 (21.6) | 86 (22.6) | 1  (24) | 0.07 (26.5) | 0.12 (26.8) | 0.13 (27) | 3.9 (27.5) | 1.9 (28.3) | 0.14 (28.4) | 0.05 (29) | 55* (30) | 50 (32) | 11 (40) | 16 (40.1) | 0.7 (40.5) | 3 (42) | 1.8 (47) | 42 (60) | Total |
| --- | --- | --- | --- | --- | --- | --- | --- | --- | --- | --- | --- | --- | --- | --- | --- | --- | --- | --- | --- | --- | --- | --- | --- | --- | --- | --- | --- | --- | --- | --- | --- |
| *Hymenophyllum plicatum* | 16.6 | 29.9 | 0.0 | 3.6 | 41.6 | 18.3 | 53.4 | 6.3 | 1.9 | 15.6 | 1.3 | 5.2 | 6.8 | 17.2 | 2.1 | 0.0 | 0.0 | 0.0 | 28.7 | 65.0 | 55.8 | 2.4 | 6.1 | 13.0 | 17.7 | 1.7 | 0.0 | 1.2 | 3.0 | 0.2 | 415 |
| *Hymenophyllum caudiculatum* | 0.3 | 2.9 | 0.0 | 2.3 | 6.9 | 17.3 | 0.0 | 17.6 | 1.0 | 0.5 | 0.0 | 1.5 | 19.9 | 87.2 | 7.7 | 0.0 | 0.0 | 0.0 | 0.0 | 3.3 | 0.0 | 0.0 | 36.7 | 37.8 | 0.0 | 0.0 | 0.0 | 0.0 | 0.0 | 0.0 | 243 |
| *Aspleium dareoides* | 6.6 | 28.7 | 0.0 | 0.7 | 21.1 | 5.1 | 23.9 | 4.9 | 12.2 | 12.3 | 0.0 | 2.3 | 2.6 | 5.4 | 4.4 | 0.0 | 0.0 | 0.0 | 0.0 | 0.6 | 0.0 | 0.0 | 1.6 | 4.6 | 0.7 | 0.4 | 0.0 | 0.7 | 3.1 | 8.8 | 151 |
| *Hymenophyllum dicranotrichum* | 9.5 | 0.0 | 0.0 | 0.0 | 0.9 | 15.4 | 2.1 | 0.0 | 1.7 | 0.0 | 0.0 | 0.0 | 10.2 | 47.1 | 0.0 | 0.0 | 0.0 | 0.0 | 0.0 | 2.9 | 0.0 | 0.0 | 12.5 | 43.0 | 0.0 | 0.0 | 0.0 | 0.0 | 0.0 | 0.0 | 145 |
| *Asplenium trilobum** | 1.8 | 12.8 | 0.1 | 0.2 | 3.4 | 0.0 | 0.0 | 0.2 | 0.0 | 7.3 | 0.8 | 3.0 | 0.9 | 12.9 | 34.2 | 0.0 | 0.0 | 22.3 | 7.4 | 8.6 | 9.2 | 3.2 | 0.0 | 0.2 | 1.7 | 0.1 | 0.0 | 4.7 | 4.7 | 1.9 | 142 |
| *Hymenophyllum krauseanum* | 10.1 | 2.7 | 0.0 | 0.5 | 33.5 | 10.7 | 7.9 | 0.2 | 5.1 | 4.8 | 0.0 | 1.4 | 12.1 | 0.4 | 7.2 | 0.0 | 0.0 | 0.0 | 0.0 | 0.0 | 0.0 | 0.0 | 5.3 | 4.1 | 12.5 | 0.1 | 0.0 | 0.0 | 1.0 | 0.3 | 120 |
| *Hymenoglossum cruentum* | 0.1 | 0.0 | 0.0 | 0.0 | 3.1 | 5.4 | 7.4 | 0.0 | 0.0 | 0.0 | 0.0 | 0.0 | 2.7 | 20.4 | 0.0 | 0.0 | 0.0 | 0.0 | 0.0 | 0.0 | 0.0 | 0.0 | 5.4 | 3.4 | 0.0 | 0.0 | 0.0 | 0.0 | 0.0 | 0.0 | 48 |
| *Hymenophyllum peltatum* | 8.3 | 1.1 | 0.0 | 0.0 | 4.0 | 2.8 | 5.0 | 0.9 | 0.3 | 0.0 | 0.0 | 0.0 | 5.7 | 0.0 | 0.0 | 0.0 | 0.0 | 0.0 | 0.0 | 0.0 | 0.0 | 0.0 | 3.6 | 4.6 | 0.0 | 0.0 | 0.0 | 0.0 | 0.0 | 0.0 | 36 |
| *Sarmienta scandens* | 1.6 | 0.3 | 0.0 | 0.1 | 0.9 | 4.6 | 0.3 | 0.5 | 0.2 | 1.5 | 0.0 | 0.1 | 1.8 | 0.2 | 0.5 | 0.0 | 0.0 | 0.0 | 0.0 | 10.3 | 0.0 | 1.9 | 0.9 | 0.8 | 2.6 | 0.5 | 0.0 | 0.9 | 0.4 | 0.7 | 32 |
| *Grammitis magellanica* | 0.1 | 0.0 | 0.0 | 0.2 | 4.8 | 0.0 | 22.3 | 0.0 | 0.0 | 0.2 | 0.0 | 0.0 | 0.1 | 0.0 | 0.0 | 0.0 | 0.0 | 0.0 | 0.0 | 0.0 | 0.0 | 0.0 | 0.0 | 0.0 | 0.0 | 0.0 | 0.0 | 0.0 | 0.0 | 0.0 | 28 |
| *Fascicularia bicolor* | 0.3 | 0.2 | 0.0 | 0.1 | 3.9 | 1.1 | 0.5 | 0.9 | 0.1 | 0.5 | 0.0 | 0.3 | 0.2 | 0.9 | 0.7 | 0.0 | 0.0 | 0.0 | 0.0 | 0.0 | 0.0 | 0.0 | 2.7 | 0.5 | 0.2 | 0.1 | 0.0 | 0.0 | 0.0 | 0.0 | 13 |
| *Polypodium feuyllei** | 0.2 | 0.0 | 0.1 | 0.0 | 0.2 | 0.1 | 0.1 | 0.0 | 0.2 | 0.1 | 0.5 | 0.0 | 0.0 | 0.0 | 0.2 | 0.6 | 0.0 | 2.8 | 1.1 | 0.1 | 4.1 | 0.4 | 0.1 | 0.0 | 0.1 | 0.4 | 0.0 | 0.0 | 0.5 | 0.5 | 12 |
| *Hymenophyllum dentatum* | 1.6 | 0.1 | 0.0 | 0.1 | 0.3 | 4.6 | 1.7 | 0.2 | 0.0 | 0.5 | 0.0 | 0.6 | 0.9 | 0.1 | 0.0 | 0.0 | 0.0 | 0.0 | 0.0 | 0.0 | 0.0 | 0.0 | 0.1 | 1.0 | 0.0 | 0.0 | 0.0 | 0.0 | 0.0 | 0.0 | 12 |
| *Hymenophyllum cuneatum* | 0.0 | 0.0 | 0.0 | 0.8 | 0.3 | 0.0 | 0.2 | 0.0 | 0.0 | 0.0 | 0.0 | 0.0 | 1.9 | 0.4 | 0.0 | 0.0 | 0.0 | 0.0 | 0.0 | 0.0 | 0.0 | 0.0 | 0.0 | 5.4 | 0.0 | 0.0 | 0.0 | 0.0 | 0.0 | 0.0 | 9 |
| *Hymenophyllum pectinatum* | 0.1 | 1.1 | 0.0 | 0.0 | 0.1 | 0.7 | 0.0 | 0.0 | 0.0 | 0.0 | 0.0 | 0.0 | 0.6 | 3.7 | 0.0 | 0.0 | 0.0 | 0.0 | 0.0 | 0.0 | 0.0 | 0.0 | 0.2 | 1.8 | 0.0 | 0.0 | 0.0 | 0.0 | 0.0 | 0.0 | 8 |
| *Hymenophyllum secundum* | 0.1 | 0.0 | 0.0 | 0.0 | 0.4 | 0.0 | 0.0 | 0.0 | 0.0 | 0.0 | 0.0 | 0.0 | 1.0 | 0.0 | 0.0 | 0.0 | 0.0 | 0.0 | 0.0 | 0.0 | 0.0 | 0.0 | 0.0 | 0.0 | 0.0 | 0.0 | 0.0 | 0.0 | 0.0 | 0.0 | 1 |
| Total | 57 | 80 | 0 | 9 | 126 | 86 | 125 | 32 | 23 | 43 | 3 | 15 | 67 | 196 | 57 | 1 | 0 | 25 | 37 | 91 | 69 | 8 | 75 | 120 | 35 | 3 | 0 | 8 | 13 | 12 |  |

1. Presence-absence matrix of climbers ordered by area.

| Especie | 936 | 156 | 133 | 86 | 67 | 55 | 50 | 42 | 40 | 33 | 22 | 16 | 11 | 9.4 | 7.1 | 5.5 | 3.9 | 3.4 | 3 | 1.9 | 1.8 | 1 | 0,8* | 0.7 | 0.15 | 0.14 | 0.13 | 0.12 | 0.07 | 0.05 | Total |
| --- | --- | --- | --- | --- | --- | --- | --- | --- | --- | --- | --- | --- | --- | --- | --- | --- | --- | --- | --- | --- | --- | --- | --- | --- | --- | --- | --- | --- | --- | --- | --- |
| *Cissus striata* | 1 | 1 | 1 | 1 | 0 | 1 | 1 | 1 | 1 | 1 | 0 | 1 | 1 | 1 | 1 | 1 | 1 | 1 | 1 | 1 | 1 | 1 | 1 | 1 | 1 | 1 | 1 | 1 | 1 | 1 | 28 |
| *Boquila trifoliolata* | 1 | 1 | 1 | 1 | 1 | 1 | 0 | 1 | 1 | 1 | 1 | 0 | 0 | 1 | 1 | 1 | 1 | 1 | 1 | 1 | 1 | 1 | 0 | 1 | 1 | 1 | 1 | 1 | 1 | 1 | 26 |
| *Luzuriaga radicans* | 1 | 1 | 1 | 1 | 1 | 1 | 1 | 1 | 1 | 1 | 1 | 1 | 1 | 1 | 1 | 1 | 1 | 1 | 1 | 1 | 1 | 1 | 0 | 1 | 0 | 1 | 0 | 1 | 0 | 0 | 25 |
| *Mitraria coccinea* | 1 | 1 | 1 | 1 | 0 | 1 | 1 | 1 | 1 | 1 | 1 | 0 | 1 | 0 | 1 | 1 | 1 | 0 | 1 | 1 | 1 | 1 | 0 | 0 | 0 | 0 | 1 | 0 | 0 | 1 | 20 |
| *Lapageria rosea* | 1 | 1 | 1 | 1 | 1 | 1 | 1 | 1 | 1 | 1 | 0 | 0 | 0 | 0 | 1 | 1 | 0 | 0 | 1 | 1 | 1 | 1 | 0 | 0 | 0 | 0 | 1 | 1 | 0 | 0 | 18 |
| *Nertera granadensis* | 1 | 1 | 1 | 1 | 1 | 1 | 1 | 0 | 1 | 1 | 0 | 0 | 0 | 1 | 1 | 1 | 1 | 0 | 1 | 0 | 1 | 0 | 0 | 1 | 0 | 1 | 1 | 0 | 0 | 0 | 18 |
| *Luzuriaga polyphylla* | 1 | 1 | 1 | 1 | 1 | 1 | 1 | 0 | 0 | 0 | 0 | 0 | 0 | 1 | 1 | 1 | 0 | 0 | 1 | 1 | 0 | 1 | 0 | 0 | 1 | 0 | 1 | 0 | 0 | 0 | 15 |
| *Muehlenbeckia hastulata** | 1 | 1 | 0 | 1 | 0 | 0 | 1 | 1 | 0 | 1 | 0 | 0 | 0 | 1 | 0 | 1 | 0 | 0 | 0 | 0 | 1 | 0 | 1 | 0 | 1 | 0 | 0 | 1 | 0 | 1 | 13 |
| *Campsidium valdivianum* | 0 | 0 | 0 | 1 | 1 | 0 | 1 | 1 | 1 | 0 | 0 | 0 | 0 | 0 | 0 | 1 | 0 | 0 | 0 | 0 | 0 | 1 | 0 | 0 | 0 | 0 | 1 | 0 | 0 | 0 | 8 |
| *Pseudopanax valdiviensis* | 0 | 1 | 1 | 1 | 0 | 1 | 1 | 0 | 0 | 0 | 0 | 0 | 0 | 0 | 0 | 1 | 0 | 0 | 1 | 0 | 0 | 1 | 0 | 0 | 0 | 1 | 0 | 0 | 0 | 0 | 9 |
| *Hydrangea serattifolia* | 0 | 0 | 1 | 1 | 0 | 1 | 0 | 0 | 1 | 0 | 0 | 0 | 0 | 0 | 0 | 0 | 0 | 0 | 0 | 0 | 0 | 0 | 0 | 0 | 0 | 0 | 0 | 0 | 0 | 0 | 4 |
| *Griselinia racemosa* | 0 | 1 | 0 | 0 | 0 | 0 | 0 | 0 | 0 | 0 | 1 | 0 | 0 | 0 | 0 | 0 | 0 | 0 | 0 | 0 | 0 | 0 | 0 | 0 | 0 | 0 | 0 | 0 | 0 | 0 | 2 |
| *Ercilla spp.** | 0 | 0 | 0 | 0 | 0 | 0 | 1 | 0 | 0 | 0 | 0 | 0 | 0 | 1 | 0 | 0 | 0 | 0 | 0 | 0 | 0 | 0 | 1 | 0 | 0 | 0 | 0 | 0 | 0 | 0 | 3 |
| *Dioscorea auriculata* | 0 | 0 | 0 | 0 | 0 | 0 | 0 | 0 | 0 | 0 | 0 | 0 | 0 | 0 | 0 | 0 | 0 | 0 | 0 | 0 | 0 | 1 | 0 | 0 | 0 | 0 | 0 | 0 | 0 | 0 | 1 |
| *Galium hypocarpium* | 0 | 0 | 1 | 0 | 0 | 0 | 0 | 0 | 0 | 0 | 0 | 0 | 0 | 0 | 0 | 0 | 0 | 0 | 0 | 0 | 0 | 0 | 0 | 0 | 0 | 0 | 0 | 0 | 0 | 0 | 1 |
| Total | 8 | 10 | 10 | 11 | 6 | 9 | 10 | 7 | 8 | 7 | 4 | 2 | 3 | 7 | 7 | 10 | 5 | 3 | 8 | 6 | 7 | 9 | 3 | 4 | 4 | 5 | 7 | 5 | 2 | 4 |  |

1. Presence-absence matrix of climbers ordered by distance.

| Especie | 936  (0) | 9.4  (8) | 0.8*  (11) | 40 (15) | 133 (15.2) | 67 (15.7) | 33  (15.8) | 22  (17) | 5.5 (17.3) | 7.1 (18) | 0.15 (21) | 3.4 (21,5) | 156 (21.6) | 86 (22.6) | 1  (24) | 0.07 (26.5) | 0.12 (26.8) | 0.13 (27) | 3.9 (27.5) | 1.9 (28.3) | 0.14 (28.4) | 0.05 (29) | 55 (30) | 50 (32) | 11 (40) | 16 (40.1) | 0.7 (40.5) | 3 (42) | 1.8 (47) | 42 (60) | Total |
| --- | --- | --- | --- | --- | --- | --- | --- | --- | --- | --- | --- | --- | --- | --- | --- | --- | --- | --- | --- | --- | --- | --- | --- | --- | --- | --- | --- | --- | --- | --- | --- |
| *Cissus striata* | 1 | 1 | 1 | 1 | 1 | 0 | 1 | 0 | 1 | 1 | 1 | 1 | 1 | 1 | 1 | 1 | 1 | 1 | 1 | 1 | 1 | 1 | 1 | 1 | 1 | 1 | 1 | 1 | 1 | 1 | 28 |
| *Boquila trifoliolata* | 1 | 1 | 0 | 1 | 1 | 1 | 1 | 1 | 1 | 1 | 1 | 1 | 1 | 1 | 1 | 1 | 1 | 1 | 1 | 1 | 1 | 1 | 1 | 0 | 0 | 0 | 1 | 1 | 1 | 1 | 26 |
| *Luzuriaga radicans* | 1 | 1 | 0 | 1 | 1 | 1 | 1 | 1 | 1 | 1 | 0 | 1 | 1 | 1 | 1 | 0 | 1 | 0 | 1 | 1 | 1 | 0 | 1 | 1 | 1 | 1 | 1 | 1 | 1 | 1 | 25 |
| *Mitraria coccinea* | 1 | 0 | 0 | 1 | 1 | 0 | 1 | 1 | 1 | 1 | 0 | 0 | 1 | 1 | 1 | 0 | 0 | 1 | 1 | 1 | 0 | 1 | 1 | 1 | 1 | 0 | 0 | 1 | 1 | 1 | 20 |
| *Lapageria rosea* | 1 | 0 | 0 | 1 | 1 | 1 | 1 | 0 | 1 | 1 | 0 | 0 | 1 | 1 | 1 | 0 | 1 | 1 | 0 | 1 | 0 | 0 | 1 | 1 | 0 | 0 | 0 | 1 | 1 | 1 | 18 |
| *Nertera granadensis* | 1 | 1 | 0 | 1 | 1 | 1 | 1 | 0 | 1 | 1 | 0 | 0 | 1 | 1 | 0 | 0 | 0 | 1 | 1 | 0 | 1 | 0 | 1 | 1 | 0 | 0 | 1 | 1 | 1 | 0 | 18 |
| *Luzuriaga polyphylla* | 1 | 1 | 0 | 0 | 1 | 1 | 0 | 0 | 1 | 1 | 1 | 0 | 1 | 1 | 1 | 0 | 0 | 1 | 0 | 1 | 0 | 0 | 1 | 1 | 0 | 0 | 0 | 1 | 0 | 0 | 15 |
| *Muehlenbeckia hastulata* | 1 | 1 | 1 | 0 | 0 | 0 | 1 | 0 | 1 | 0 | 1 | 0 | 1 | 1 | 0 | 0 | 1 | 0 | 0 | 0 | 0 | 1 | 0 | 1 | 0 | 0 | 0 | 0 | 1 | 1 | 13 |
| *Campsidium valdivianum* | 0 | 0 | 0 | 1 | 0 | 1 | 0 | 0 | 1 | 0 | 0 | 0 | 0 | 1 | 1 | 0 | 0 | 1 | 0 | 0 | 0 | 0 | 0 | 1 | 0 | 0 | 0 | 0 | 0 | 1 | 8 |
| *Pseudopanax valdiviensis* | 0 | 0 | 0 | 0 | 1 | 0 | 0 | 0 | 1 | 0 | 0 | 0 | 1 | 1 | 1 | 0 | 0 | 0 | 0 | 0 | 1 | 0 | 1 | 1 | 0 | 0 | 0 | 1 | 0 | 0 | 9 |
| *Hydrangea serattifolia* | 0 | 0 | 0 | 1 | 1 | 0 | 0 | 0 | 0 | 0 | 0 | 0 | 0 | 1 | 0 | 0 | 0 | 0 | 0 | 0 | 0 | 0 | 1 | 0 | 0 | 0 | 0 | 0 | 0 | 0 | 4 |
| *Griselinia racemosa* | 0 | 0 | 0 | 0 | 0 | 0 | 0 | 1 | 0 | 0 | 0 | 0 | 1 | 0 | 0 | 0 | 0 | 0 | 0 | 0 | 0 | 0 | 0 | 0 | 0 | 0 | 0 | 0 | 0 | 0 | 2 |
| *Ercilla spp.* | 0 | 1 | 1 | 0 | 0 | 0 | 0 | 0 | 0 | 0 | 0 | 0 | 0 | 0 | 0 | 0 | 0 | 0 | 0 | 0 | 0 | 0 | 0 | 1 | 0 | 0 | 0 | 0 | 0 | 0 | 3 |
| *Dioscorea auriculata* | 0 | 0 | 0 | 0 | 0 | 0 | 0 | 0 | 0 | 0 | 0 | 0 | 0 | 0 | 1 | 0 | 0 | 0 | 0 | 0 | 0 | 0 | 0 | 0 | 0 | 0 | 0 | 0 | 0 | 0 | 1 |
| *Galium hypocarpium* | 0 | 0 | 0 | 0 | 1 | 0 | 0 | 0 | 0 | 0 | 0 | 0 | 0 | 0 | 0 | 0 | 0 | 0 | 0 | 0 | 0 | 0 | 0 | 0 | 0 | 0 | 0 | 0 | 0 | 0 | 1 |
| Total | 8 | 7 | 3 | 8 | 10 | 6 | 7 | 4 | 10 | 7 | 4 | 3 | 10 | 11 | 9 | 2 | 5 | 7 | 5 | 6 | 5 | 4 | 9 | 10 | 3 | 2 | 4 | 8 | 7 | 7 |  |

1. Quantitative matrix of climbers ordered by area.

| Especie | 936 | 156 | 133 | 86 | 67 | 55 | 50 | 42 | 40 | 33 | 22* | 16 | 11 | 9.4 | 7.1 | 5.5 | 3.9 | 3.4 | 3 | 1.9 | 1.8 | 1 | 0,8* | 0.7 | 0.15 | 0.14 | 0.13 | 0.12 | 0.07 | 0,05* | Total |
| --- | --- | --- | --- | --- | --- | --- | --- | --- | --- | --- | --- | --- | --- | --- | --- | --- | --- | --- | --- | --- | --- | --- | --- | --- | --- | --- | --- | --- | --- | --- | --- |
| *Luzuriaga radicans* | 1.65 | 2.90 | 4.26 | 5.33 | 0.32 | 3.74 | 1.94 | 0.16 | 3.61 | 1.08 | 0.57 | 0.03 | 0.23 | 0.90 | 4.83 | 0.11 | 0.76 | 1.84 | 0.03 | 0.14 | 0.74 | 0.72 | 0.00 | 0.59 | 0.00 | 0.15 | 0.00 | 0.11 | 0.00 | 0.00 | 36.8 |
| *Cissus striata** | 0.09 | 0.30 | 0.00 | 12.28 | 0.00 | 0.33 | 0.30 | 1.56 | 2.39 | 0.29 | 0.00 | 0.32 | 0.65 | 0.05 | 1.75 | 1.36 | 0.21 | 0.42 | 0.46 | 0.43 | 0.41 | 2.22 | 0.06 | 0.05 | 0.33 | 0.77 | 0.05 | 0.33 | 0.21 | 1.25 | 28.9 |
| *Mitraria coccinea* | 0.44 | 0.85 | 1.35 | 0.44 | 0.00 | 0.63 | 0.60 | 0.12 | 0.09 | 0.75 | 0.05 | 0.00 | 0.04 | 0.00 | 0.42 | 0.33 | 0.03 | 0.00 | 2.54 | 1.00 | 0.21 | 0.50 | 0.00 | 0.00 | 0.00 | 0.00 | 0.05 | 0.00 | 0.00 | 1.38 | 11.8 |
| *Boquila trifoliolata* | 0.20 | 0.22 | 0.35 | 0.28 | 0.26 | 0.15 | 0.02 | 1.77 | 0.45 | 0.25 | 0.05 | 0.00 | 0.00 | 0.10 | 0.08 | 0.83 | 0.21 | 0.03 | 0.11 | 0.29 | 0.03 | 0.72 | 0.00 | 0.59 | 0.25 | 0.31 | 0.05 | 0.04 | 0.21 | 0.06 | 7.9 |
| *Luzuriaga polyphylla* | 0.76 | 0.19 | 0.21 | 0.56 | 0.79 | 0.04 | 0.80 | 0.00 | 0.00 | 0.00 | 0.00 | 0.00 | 0.00 | 0.33 | 0.04 | 0.22 | 0.00 | 0.00 | 0.23 | 0.71 | 0.00 | 0.06 | 0.00 | 0.00 | 0.00 | 0.00 | 0.09 | 0.00 | 0.00 | 0.00 | 5.0 |
| *Lapageria rosea* | 0.05 | 0.04 | 0.03 | 1.06 | 0.05 | 0.02 | 0.04 | 0.01 | 0.06 | 0.17 | 0.00 | 0.00 | 0.00 | 0.00 | 0.08 | 0.03 | 0.00 | 0.00 | 0.03 | 0.14 | 0.03 | 0.06 | 0.00 | 0.00 | 0.00 | 0.00 | 0.14 | 0.04 | 0.00 | 0.00 | 2.1 |
| *Nertera granadensis* | 0.01 | 0.01 | 0.62 | 0.06 | 0.05 | 0.02 | 0.04 | 0.00 | 0.03 | 0.04 | 0.00 | 0.00 | 0.00 | 0.05 | 0.04 | 0.03 | 0.03 | 0.00 | 0.03 | 0.00 | 0.03 | 0.00 | 0.00 | 0.05 | 0.00 | 0.08 | 0.05 | 0.00 | 0.00 | 0.00 | 1.3 |
| *Campsidium valdivianum* | 0.00 | 0.00 | 0.00 | 0.06 | 0.05 | 0.00 | 0.16 | 0.01 | 0.03 | 0.00 | 0.00 | 0.00 | 0.00 | 0.00 | 0.00 | 0.17 | 0.00 | 0.00 | 0.00 | 0.00 | 0.00 | 0.06 | 0.00 | 0.00 | 0.00 | 0.00 | 0.09 | 0.00 | 0.00 | 0.00 | 0.6 |
| *Pseudopanax valdiviensis* | 0.00 | 0.01 | 0.03 | 0.06 | 0.00 | 0.07 | 0.06 | 0.00 | 0.00 | 0.00 | 0.00 | 0.00 | 0.00 | 0.00 | 0.00 | 0.00 | 0.00 | 0.00 | 0.03 | 0.00 | 0.00 | 0.22 | 0.00 | 0.00 | 0.00 | 0.08 | 0.00 | 0.00 | 0.00 | 0.00 | 0.6 |
| *Muehlenbeckia hastulata* | 0.01 | 0.01 | 0.00 | 0.06 | 0.00 | 0.00 | 0.02 | 0.01 | 0.00 | 0.04 | 0.00 | 0.00 | 0.00 | 0.05 | 0.00 | 0.03 | 0.00 | 0.00 | 0.00 | 0.00 | 0.03 | 0.00 | 0.06 | 0.00 | 0.00 | 0.00 | 0.00 | 0.04 | 0.00 | 0.06 | 0.4 |
| *Hydrangea serattifolia* | 0.00 | 0.00 | 0.03 | 0.22 | 0.00 | 0.04 | 0.00 | 0.00 | 0.03 | 0.00 | 0.00 | 0.00 | 0.00 | 0.00 | 0.00 | 0.00 | 0.00 | 0.00 | 0.00 | 0.00 | 0.00 | 0.00 | 0.00 | 0.00 | 0.00 | 0.00 | 0.00 | 0.00 | 0.00 | 0.00 | 0.3 |
| *Ercilla spp.** | 0.00 | 0.00 | 0.00 | 0.00 | 0.00 | 0.00 | 0.02 | 0.00 | 0.00 | 0.00 | 0.00 | 0.00 | 0.00 | 0.05 | 0.00 | 0.00 | 0.00 | 0.00 | 0.00 | 0.00 | 0.00 | 0.00 | 0.06 | 0.00 | 0.00 | 0.00 | 0.00 | 0.00 | 0.00 | 0.00 | 0.1 |
| *Griselinia racemosa* | 0.00 | 0.04 | 0.00 | 0.00 | 0.00 | 0.00 | 0.00 | 0.00 | 0.00 | 0.00 | 0.05 | 0.00 | 0.00 | 0.00 | 0.00 | 0.00 | 0.00 | 0.00 | 0.00 | 0.00 | 0.00 | 0.00 | 0.00 | 0.00 | 0.00 | 0.00 | 0.00 | 0.00 | 0.00 | 0.00 | 0.1 |
| *Dioscorea auriculata* | 0.00 | 0.00 | 0.00 | 0.00 | 0.00 | 0.00 | 0.00 | 0.00 | 0.00 | 0.00 | 0.00 | 0.00 | 0.00 | 0.00 | 0.00 | 0.00 | 0.00 | 0.00 | 0.00 | 0.00 | 0.00 | 0.06 | 0.00 | 0.00 | 0.00 | 0.00 | 0.00 | 0.00 | 0.00 | 0.00 | 0.1 |
| *Galium hypocarpium* | 0.00 | 0.00 | 0.03 | 0.00 | 0.00 | 0.00 | 0.00 | 0.00 | 0.00 | 0.00 | 0.00 | 0.00 | 0.00 | 0.00 | 0.00 | 0.00 | 0.00 | 0.00 | 0.00 | 0.00 | 0.00 | 0.00 | 0.00 | 0.00 | 0.00 | 0.00 | 0.00 | 0.00 | 0.00 | 0.00 | 0.0 |
| Total | 3.2 | 4.6 | 6.9 | 20.4 | 1.5 | 5.0 | 4.0 | 3.6 | 6.7 | 2.6 | 0.7 | 0.4 | 0.9 | 1.5 | 7.3 | 3.1 | 1.2 | 2.3 | 3.5 | 2.7 | 1.5 | 4.6 | 0.2 | 1.3 | 0.6 | 1.4 | 0.5 | 0.6 | 0.4 | 2.8 |  |

Quantitative matrix of climbers ordered by distance.

| Especie | 936  (0) | 9.4  (8) | 0.8*  (11) | 40 (15) | 133* (15.2) | 67 (15.7) | 33  (15.8) | 22*  (17) | 5.5 (17.3) | 7.1 (18) | 0.15 (21) | 3.4 (21,5) | 156 (21.6) | 86 (22.6) | 1  (24) | 0.07 (26.5) | 0.12 (26.8) | 0.13 (27) | 3.9 (27.5) | 1.9 (28.3) | 0.14 (28.4) | 0.05 (29) | 55 (30) | 50 (32) | 11 (40) | 16 (40.1) | 0.7 (40.5) | 3 (42) | 1.8 (47) | 42 (60) | Total |
| --- | --- | --- | --- | --- | --- | --- | --- | --- | --- | --- | --- | --- | --- | --- | --- | --- | --- | --- | --- | --- | --- | --- | --- | --- | --- | --- | --- | --- | --- | --- | --- |
| *Luzuriaga radicans* | 1.65 | 0.90 | 0.00 | 3.61 | 4.26 | 0.32 | 1.08 | 0.57 | 0.11 | 4.83 | 0.00 | 1.84 | 2.90 | 5.3 | 0.72 | 0.00 | 0.11 | 0.00 | 0.76 | 0.14 | 0.15 | 0.00 | 3.74 | 1.94 | 0.23 | 0.03 | 0.59 | 0.03 | 0.74 | 0.16 | 36.8 |
| *Cissus striata** | 0.09 | 0.05 | 0.06 | 2.39 | 0.00 | 0.00 | 0.29 | 0.00 | 1.36 | 1.75 | 0.33 | 0.42 | 0.30 | 12.3 | 2.22 | 0.21 | 0.33 | 0.05 | 0.21 | 0.43 | 0.77 | 1.25 | 0.33 | 0.30 | 0.65 | 0.32 | 0.05 | 0.46 | 0.41 | 1.56 | 28.9 |
| *Mitraria coccinea* | 0.44 | 0.00 | 0.00 | 0.09 | 1.35 | 0.00 | 0.75 | 0.05 | 0.33 | 0.42 | 0.00 | 0.00 | 0.85 | 0.44 | 0.50 | 0.00 | 0.00 | 0.05 | 0.03 | 1.00 | 0.00 | 1.38 | 0.63 | 0.60 | 0.04 | 0.00 | 0.00 | 2.54 | 0.21 | 0.12 | 11.8 |
| *Boquila trifoliolata* | 0.20 | 0.10 | 0.00 | 0.45 | 0.35 | 0.26 | 0.25 | 0.05 | 0.83 | 0.08 | 0.25 | 0.03 | 0.22 | 0.28 | 0.72 | 0.21 | 0.04 | 0.05 | 0.21 | 0.29 | 0.31 | 0.06 | 0.15 | 0.02 | 0.00 | 0.00 | 0.59 | 0.11 | 0.03 | 1.77 | 7.9 |
| *Luzuriaga polyphylla* | 0.76 | 0.33 | 0.00 | 0.00 | 0.21 | 0.79 | 0.00 | 0.00 | 0.22 | 0.04 | 0.00 | 0.00 | 0.19 | 0.56 | 0.06 | 0.00 | 0.00 | 0.09 | 0.00 | 0.71 | 0.00 | 0.00 | 0.04 | 0.80 | 0.00 | 0.00 | 0.00 | 0.23 | 0.00 | 0.00 | 5.0 |
| *Lapageria rosea* | 0.05 | 0.00 | 0.00 | 0.06 | 0.03 | 0.05 | 0.17 | 0.00 | 0.03 | 0.08 | 0.00 | 0.00 | 0.04 | 1.06 | 0.06 | 0.00 | 0.04 | 0.14 | 0.00 | 0.14 | 0.00 | 0.00 | 0.02 | 0.04 | 0.00 | 0.00 | 0.00 | 0.03 | 0.03 | 0.01 | 2.1 |
| *Nertera granadensis* | 0.01 | 0.05 | 0.00 | 0.03 | 0.62 | 0.05 | 0.04 | 0.00 | 0.03 | 0.04 | 0.00 | 0.00 | 0.01 | 0.06 | 0.00 | 0.00 | 0.00 | 0.05 | 0.03 | 0.00 | 0.08 | 0.00 | 0.02 | 0.04 | 0.00 | 0.00 | 0.05 | 0.03 | 0.03 | 0.00 | 1.3 |
| *Campsidium valdivianum* | 0.00 | 0.00 | 0.00 | 0.03 | 0.00 | 0.05 | 0.00 | 0.00 | 0.17 | 0.00 | 0.00 | 0.00 | 0.00 | 0.06 | 0.06 | 0.00 | 0.00 | 0.09 | 0.00 | 0.00 | 0.00 | 0.00 | 0.00 | 0.16 | 0.00 | 0.00 | 0.00 | 0.00 | 0.00 | 0.01 | 0.6 |
| *Pseudopanax valdiviensis* | 0.00 | 0.00 | 0.00 | 0.00 | 0.03 | 0.00 | 0.00 | 0.00 | 0.00 | 0.00 | 0.00 | 0.00 | 0.01 | 0.06 | 0.22 | 0.00 | 0.00 | 0.00 | 0.00 | 0.00 | 0.08 | 0.00 | 0.07 | 0.06 | 0.00 | 0.00 | 0.00 | 0.03 | 0.00 | 0.00 | 0.6 |
| *Muehlenbeckia hastulata* | 0.01 | 0.05 | 0.06 | 0.00 | 0.00 | 0.00 | 0.04 | 0.00 | 0.03 | 0.00 | 0.00 | 0.00 | 0.01 | 0.06 | 0.00 | 0.00 | 0.04 | 0.00 | 0.00 | 0.00 | 0.00 | 0.06 | 0.00 | 0.02 | 0.00 | 0.00 | 0.00 | 0.00 | 0.03 | 0.01 | 0.4 |
| *Hydrangea serattifolia* | 0.00 | 0.00 | 0.00 | 0.03 | 0.03 | 0.00 | 0.00 | 0.00 | 0.00 | 0.00 | 0.00 | 0.00 | 0.00 | 0.22 | 0.00 | 0.00 | 0.00 | 0.00 | 0.00 | 0.00 | 0.00 | 0.00 | 0.04 | 0.00 | 0.00 | 0.00 | 0.00 | 0.00 | 0.00 | 0.00 | 0.3 |
| *Ercilla spp.* | 0.00 | 0.05 | 0.06 | 0.00 | 0.00 | 0.00 | 0.00 | 0.00 | 0.00 | 0.00 | 0.00 | 0.00 | 0.00 | 0.00 | 0.00 | 0.00 | 0.00 | 0.00 | 0.00 | 0.00 | 0.00 | 0.00 | 0.00 | 0.02 | 0.00 | 0.00 | 0.00 | 0.00 | 0.00 | 0.00 | 0.1 |
| *Griselinia racemosa* | 0.00 | 0.00 | 0.00 | 0.00 | 0.00 | 0.00 | 0.00 | 0.05 | 0.00 | 0.00 | 0.00 | 0.00 | 0.04 | 0.00 | 0.00 | 0.00 | 0.00 | 0.00 | 0.00 | 0.00 | 0.00 | 0.00 | 0.00 | 0.00 | 0.00 | 0.00 | 0.00 | 0.00 | 0.00 | 0.00 | 0.1 |
| *Dioscorea auriculata* | 0.00 | 0.00 | 0.00 | 0.00 | 0.00 | 0.00 | 0.00 | 0.00 | 0.00 | 0.00 | 0.00 | 0.00 | 0.00 | 0.00 | 0.06 | 0.00 | 0.00 | 0.00 | 0.00 | 0.00 | 0.00 | 0.00 | 0.00 | 0.00 | 0.00 | 0.00 | 0.00 | 0.00 | 0.00 | 0.00 | 0.1 |
| *Galium hypocarpium* | 0.00 | 0.00 | 0.00 | 0.00 | 0.03 | 0.00 | 0.00 | 0.00 | 0.00 | 0.00 | 0.00 | 0.00 | 0.00 | 0.00 | 0.00 | 0.00 | 0.00 | 0.00 | 0.00 | 0.00 | 0.00 | 0.00 | 0.00 | 0.00 | 0.00 | 0.00 | 0.00 | 0.00 | 0.00 | 0.00 | 0.0 |
| Total | 3.2 | 1.5 | 0.2 | 6.7 | 6.9 | 1.5 | 2.6 | 0.7 | 3.1 | 7.3 | 0.6 | 2.3 | 4.6 | 20.4 | 4.6 | 0.4 | 0.6 | 0.5 | 1.2 | 2.7 | 1.4 | 2.8 | 5.0 | 4.0 | 0.9 | 0.4 | 1.3 | 3.5 | 1.5 | 3.6 |  |
